# Spatial AI in cancer: mapping immune evasion topology through multi-modal omics and deep learning

**DOI:** 10.3389/fonc.2026.1762907

**Published:** 2026-04-15

**Authors:** Lang Lang, Yuhan Cui, Haimei Wang, Yan Xiao

**Affiliations:** 1 School of Medical Sciences, Xi’an Peihua University, Xi’an, Shaanxi, China; 2 Shenzhen Technology University, Shenzhen, China; 3 The First Affiliated Hospital of Shenzhen University, Shenzhen, China; 4 Medical Innovation Technology Transformation Center of Shenzhen Second People’s Hospital, Shenzhen University, Shenzhen, China; 5 Guangzhou College of Technology and Business, Guangzhou, Guangdong, China; 6 Guangzhou Guanhong New Materials Co., Ltd., Guangzhou, China

**Keywords:** AI, deep learning, immune evasion, immunotherapy resistance, spatial transcriptomics, tumor microenvironment

## Abstract

Immune checkpoint blockade has transformed cancer therapy, achieving lasting responses in some patients, yet most still encounter primary or acquired resistance. Recent evidence demonstrates that this resistance is driven not only by intrinsic cellular features but also by the spatial organization of the tumor microenvironment (TME), including physical barriers, localized immunosuppressive niches, and organized immune cell aggregates that collectively regulate anti-tumor immunity. This review synthesizes advances in Spatial AI, combining high-resolution spatial multi-omics with deep learning approaches, particularly graph neural networks (GNNs), to elucidate the topological mechanisms of immune evasion and inform therapeutic development. Technological platforms enabling spatial molecular mapping, tools for multi-modal alignment and normalization, and computational frameworks for graph-based TME representation are covered. We define spatial phenotypes associated with immune resistance, such as immune exclusion, dysfunctional inflamed regions, and maturation states of tertiary lymphoid structures, and demonstrate how Spatial AI generates interpretable topological biomarkers that surpass conventional assays. The discussion addresses translational pathways for spatial biomarker validation and highlights key obstacles, including data standardization, computational scalability, explainability, and regulatory approval. Ultimately, immune evasion is a topological challenge, and Spatial AI offers a robust computational solution to translate complex spatial data into actionable clinical strategies to overcome architectural resistance in cancer immunotherapy.

## 1 Introduction

The advent of immune-checkpoint blockade (ICB) therapies targeting PD-1, PD-L1, and CTLA-4 has revolutionized cancer treatment by achieving durable responses in subsets of patients across melanoma, non–small cell lung cancer (NSCLC), renal cell carcinoma, and other malignancies. Landmark trials such as KEYNOTE-001 and CheckMate-067 established that reactivation of exhausted T cells can mediate long-term control even in metastatic disease (1–3). However, despite these breakthroughs, only a minority of patients experience lasting benefits, while many exhibit primary or acquired resistance. The limitations of current biomarkers, including PD-L1 expression, tumor mutational burden, or microsatellite instability, underscore the need for deeper mechanistic insights. These conventional predictors often fail because they treat tumors as molecularly homogeneous entities, neglecting the complex spatial organization that governs immune engagement and suppression (4–6).

**Figure 1:**
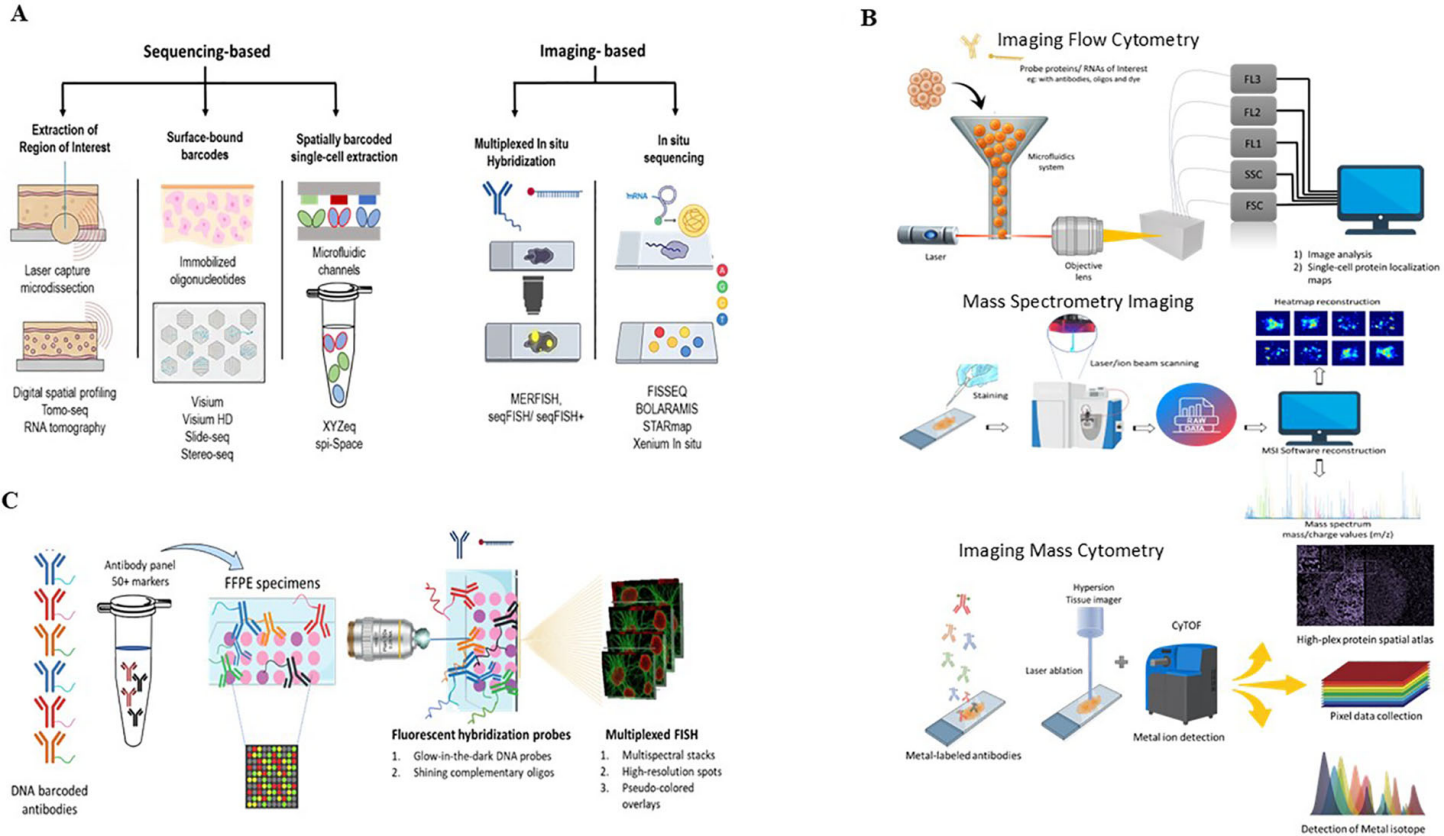
Types of spatial methods. **(A)** Sequencing-based spatial transcriptomics captures barcoded RNA for NGS readout, whereas imaging-based methods visualize transcripts through multiplexed *in situ* imaging. **(B)** Spatial proteomics maps protein levels and locations using multiplexed antibodies or mass-spectrometry-based signals. **(C)** Multiplexed spatial profiling measures numerous RNA or protein targets within tissue using barcoded or imaging-based assays.

Emerging evidence from single-cell and spatial multi-omics studies emphasizes that the tumor microenvironment (TME) behaves as an organized ecosystem shaped by both molecular and geometric constraints. The relative positioning of immune cells, stromal barriers, and vasculature dictates nutrient gradients, cytokine diffusion, and lymphocyte infiltration patterns (7–9). Non-random localization of macrophages, fibroblasts, and T cells molds immunosuppressive niches, forming exclusion zones around cancer cell clusters that block cytotoxic T-cell access. Technologies such as spatial transcriptomics, imaging mass cytometry, multiplexed ion-beam imaging, and spatial proteomics have now revealed that these spatial configurations critically determine therapeutic response (**Figure 1**) (10–13). Unlike bulk sequencing, which averages signals across all cell types or dissociative single-cell techniques, which lose the positional context necessary to interpret immune dynamics, spatial omics retains topology information. This enables the discovery of emergent resistance circuits that operate through physical and cytological organization rather than gene expression alone.

The integration of spatial multi-omics with artificial intelligence (AI) has given rise to a transformative frontier termed Spatial AI. This framework couples high-dimensional spatial data with geometric deep-learning algorithms, particularly graph neural networks (GNNs that can model cell-cell relationships in a spatially aware manner (14–18). Instead of relying on pixel-based correlations, GNNs represent tissue architecture as biological graphs, where nodes correspond to cells or regions and edges represent physical proximity or ligand–receptor interactions. These models can infer how local cellular arrangements, such as cancer-associated fibroblast (CAF) borders encasing T cells or tumor-associated macrophage (TAM)–T cell adjacency networks, predict immune dysfunction. The introduction of spatial attention mechanisms and graph transformers has further enhanced explainability, providing interpretable topological biomarkers that rival or surpass traditional molecular signatures (19, 20).

The inverse problem in spatial biology: Spatial transcriptomics fundamentally poses an inverse problem, inferring latent tissue organization from noisy, incomplete, and resolution-limited molecular measurements (21). Framing spatial biology this way clarifies why AI has become indispensable. In the forward process, tissue architecture, comprising cell states, spatial neighborhoods, and signaling gradients, generates measurable molecular profiles through a sequence of tissue organization, measurement, and observed data. However, the measurement process introduces distortions from limited spatial resolution, technical noise, and systematic biases, producing data that only partially reflect underlying biology. The inverse problem, therefore, requires reconstructing tissue architecture from degraded signals (22). This challenge is intrinsically ill-posed, as different tissue configurations can yield similar observations, particularly when spatial resolution is coarse, gene panels are incomplete, or biological signals are obscured by technical variance. Spatial omics comprises multiple such inverse problems, including denoising and deconvolution to recover true signals, segmentation and registration to delineate tissue domains, spatial niche discovery to identify cellular neighborhoods, interaction inference to reconstruct signaling networks, outcome prediction to link tissue topology to clinical phenotypes, and counterfactual modeling to simulate perturbations such as fibroblast depletion or immune niche enhancement. Classical statistical approaches often fail here due to their reliance on handcrafted features and restrictive assumptions. AI, especially deep learning, provides data-driven regularization by learning statistical priors and hierarchical representations directly from large multimodal datasets. Convolutional networks model local spatial patterns, graph neural networks capture cell–cell relationships, attention mechanisms weigh spatial context, and generative models enable predictive simulations (23). These learned priors constrain otherwise underdetermined inferences, yielding biologically plausible reconstructions. Thus, “Spatial AI” is not merely deep learning applied to spatial data, but a principled framework where learned priors regularize the inversion of complex biological processes, enabling robust discovery in spatial biology.

This review synthesizes recent advances underlying the emergence of Spatial AI as a conceptual and technological framework for decoding spatial immune resistance. It identifies three central domains advancing this field. First, we detail spatial data sources ranging from spatial transcriptomics (10x Genomics Visium, MERFISH) to multiplex imaging (CODEX, MIBI, IMC) and histomorphological alignment strategies that allow cross-modal data fusion (24–26). Second, we describe spatial phenotypes of immune evasion, including immune exclusion, dysfunctional inflamed niches, and tertiary lymphoid structure (TLS) maturation states that influence response to ICB (27–29). Finally, we explore computational frameworks for representing tissue topology through graphs, leveraging GNNs, diffusion models, and explainable AI for biomarker discovery and clinical translation. By mapping immune evasion as a topological problem, Spatial AI offers a roadmap to identify new therapeutic vulnerabilities that lie not in genes but in geometry.

## 2 Technological pillars: capturing the tumor microenvironment in space

Mapping the spatial architecture of immune evasion requires technologies capable of maintaining the physical context of tissues while resolving molecular and cellular features at high resolution. Spatial omics platforms, spanning transcriptomic, proteomic, and imaging modalities, now enable comprehensive visualization of tumor–immune interactions *in situ*. In this section, the latest developments in spatial transcriptomic and proteomic technologies are reviewed, highlighting their technical trade-offs, benchmarking efforts, and computational strategies for multimodal integration in cancer research.

### 2.1 Spatial transcriptomics: sequencing-based versus imaging-based platforms

#### 2.1.1 Sequencing-based approaches

The 10x Genomics Visium platform, characterized by approximately 55 µm spot diameter encompassing 5–20 cells per capture zone, continues to serve as a key framework for spatial transcriptome profiling with genome-wide coverage and reproducible spatial fidelity (30). In 2024, the introduction of Visium HD significantly extended these capabilities by achieving near single-cell spatial resolution in FFPE (formalin-fixed paraffin-embedded) tissues, thereby bridging the gap between histological architecture and molecular precision (**Figure 1A**) (31). The increased spot density and improved transcript capture efficiency enable more accurate cell-type deconvolution and finer delineation of transcriptional gradients across tumor invasive fronts (32).

Comparative benchmarking studies have refined the understanding of optimal sample handling and protocol design. The SpatialBenchVisium project systematically assessed performance across fresh frozen and FFPE samples using either manual or CytAssist-based placements (33). Tissues processed with probe-based hybridization and CytAssist placement demonstrated higher quality features, including improved signal-to-noise ratio and better recovery of histologically coherent immune cell structures. Cross-platform evaluations integrating Visium HD, CosMx, Xenium, and Stereo-seq v1.3 across three major cancer types (colon, hepatocellular, and ovarian) revealed complementary strengths. Imaging-based situ systems such as CosMx and Xenium offer subcellular precision with high localization accuracy, whereas sequencing-based Visium HD provides broader transcriptome coverage and more uniform spot-to-spot sensitivity distribution (34, 35).

At the computational level, spatially variable gene (SVG) detection remains pivotal for identifying microenvironmental patterns. A comprehensive benchmarking study evaluating fourteen SVG algorithms across ninety-six spatial transcriptomic datasets reported that no single method dominates across all experimental contexts. Algorithmic performance is highly dependent on spatial resolution, tissue preservation quality, and the biological question addressed, such as differential expression between tumor cores and invasive margins (32, 36). Collectively, these findings reinforce the need for platform-specific computational optimization to reliably decode the molecular geography of tumor immune evasion.

#### 2.1.2 Imaging-based and *in situ* approaches

Imaging-based spatial transcriptomic (iST) platforms have redefined transcript localization by achieving single-molecule resolution. Technologies including Xenium, CosMx, and MERSCOPE (MERFISH implementation) use cyclic labeling and optical decoding to localize individual transcripts within subcellular domains, enabling simultaneous detection of hundreds to thousands of genes per section. Their high multiplexing density allows exploration of cell-state heterogeneity, spatial gene gradients, and rare immune stromal interactions with unprecedented accuracy (**Figure 1A**) (37, 38). Comparative evaluations indicate that Xenium achieves higher transcript counts per gene and lower background noise than other *in situ* platforms, while maintaining comparable specificity (39).

Until recently, the dependence on fresh-frozen tissue limited the clinical translation of imaging-based spatial transcriptomics. However, technological advances have now extended compatibility to FFPE specimens, a critical step for deploying these tools in large clinical biobanks and retrospective cohort studies. Platforms such as Visium HD, Xenium, and CosMx have demonstrated FFPE performance comparable to fresh-frozen preparations, and cross-validation with single-cell RNA sequencing confirms high biological fidelity (21, 40, 41). This transition underscores the growing clinical readiness of spatial omics workflows.

### 2.2 Spatial proteomics and multiplexed imaging

Spatial proteomic technologies complement transcriptomics by capturing functional protein states and post-translational modifications within tissue architecture. Untargeted spatial proteomics, primarily mass spectrometry (MS) based, enables unbiased analysis of protein expression and localization without predefined targets, allowing broad proteome coverage for biomarker discovery (42, 43). In LC-MS workflows, proteins can be analyzed through bottom-up approaches, where proteins are enzymatically digested into peptides and analyzed via LC–MS/MS or top-down approaches, which examine intact proteins to preserve proteoform and modification information (44). Spatial context can be retained using laser capture microdissection (LCM), allowing region- or cell-specific profiling in heterogeneous tissues (**Figure 1B**) (45). Imaging mass spectrometry (IMS), especially MALDI-IMS, provides direct visualization of protein distributions *in situ* at spatial resolutions of 5–20 µm (46). Standard workflows involve tissue sectioning, on-tissue digestion, and matrix application using organic matrices such as α-cyano-4-hydroxycinnamic acid or sinapinic acid (47). Recent innovations such as MALDI-2 have improved ionization efficiency, sensitivity, and spatial resolution below 1 µm (48). IMS has identified clinically relevant biomarkers, including HSPB1, hnRNPA2B1, and filamin, associated with metastasis, therapy resistance, and poor prognosis in cancer (49). Although limited by acquisition time, MS/MS depth, and spatial resolution, untargeted spatial proteomics remains a powerful approach for decoding tumor heterogeneity and uncovering new therapeutic targets (50, 51).

Microscopy is now a powerful tool for three-dimensional anatomic profiling down to single-cell resolution, essential for visualizing cellular components and mapping complex tissue architecture. Historically, traditional immunofluorescence was severely restricted, typically allowing the detection of only four markers due to spectral overlap. However, recent breakthroughs in multiplex tissue imaging have shattered this barrier, enabling the simultaneous spatial detection of over 50 cellular proteins and up to 100 RNA markers at single-cell resolution (**Figure 1C**) (52). This expanded capacity is crucial for localizing multiple immune, stromal, and epithelial cell types and subsets, ultimately facilitating the detailed mapping of tissue architecture and the characterization of multicellular interactions. These advanced methods include platforms like Imaging Mass Cytometry (IMC) and Multiplexed Ion Beam Imaging (MIBI), which use metal-tagged antibodies for single-round acquisition (53), as well as cyclic methods like Co-Detection-by-InDEXing (CODEX) (54), which employ iterative cycles of fluorescent oligonucleotide binding, imaging, and stripping from DNA-barcoded antibodies. These cyclic multiplexed approaches, which combine fluorescent hybridization probes with DNA-barcoded antibodies to achieve simultaneous detection of numerous RNA and protein targets, are illustrated schematically in **Figure 1C**. The high-dimensional data generated by these techniques necessitate equally advanced bioinformatic methods for robust cell segmentation, accurate marker quantification, and sophisticated spatial analysis to derive meaningful biological and clinical insights (55, 56).

Recent cancer-specific studies illustrate the therapeutic potential of these methodologies. IMC analysis of diffuse large B-cell lymphoma identified phosphorylated S6 (p-S6), a marker of mTOR pathway activation, as both prognostic and predictive; patients exhibiting elevated p-S6 within resistant microdomains had significantly poorer outcomes, implicating active translational signaling as a hallmark of therapeutic refractoriness (57). In osteosarcoma, IMC delineated macrophage clusters characterized by CD163+ and CD206+ phenotypes that spatially co-localized with invasive tumor margins, correlating with metastatic dissemination and adverse prognosis (58, 59). These results collectively emphasize that the situ topography of immune stromal composition, rather than bulk transcript expression alone, critically determines oncologic behavior.

### 2.3 Trade-offs, quality considerations, and analytical implications

The fidelity of spatial omics data fundamentally depends on pre-analytical variables such as tissue composition, fixation quality, sectioning thickness, and library preparation strategy (60–62). Analyses from the SpatialBenchVisium dataset highlight that FFPE sections processed with optimized probe chemistry and the CytAssist alignment retain higher detection sensitivity for low-abundance transcripts, particularly immune regulatory genes (35, 63, 64).

Resolution, transcriptomic coverage, and cost balance remain central design constraints. Sequencing-based platforms, including Visium HD and Stereo seq, deliver broad, unbiased transcriptome coverage but inherently average signals from multiple neighboring cells (65–67). Imaging-based systems, while providing single-cell or sub-cellular accuracy, are restricted by predefined probe panels and may under-represent rare or unannotated transcripts (68, 69). Even with enhanced density in Visium HD, the trade-off between increased spatial resolution and modest per-spot UMI counts persists, particularly in highly heterogeneous tumor tissues.

Multiplex spatial proteomics expands interpretative capacity but introduces practical limitations related to throughput, reagent cost, and antibody validation. Techniques such as Imaging Mass Cytometry (IMC), Multiplexed Ion Beam Imaging (MIBI), and CODEX demand extensive calibration to ensure reproducibility, and imaging acquisition time limits scalability (70–72). Despite these constraints, integrative transcriptome proteome approaches provide complementary insight, since mRNA abundance does not necessarily predict functional protein activation or post-translational modification patterns (73, 74).

Sequencing-based platforms (Visium HD, Stereo-seq) represent the appropriate first-line choice when relevant cell states, gene programs, or spatial signatures remain undefined. Their defining advantage, unbiased genome-wide transcriptomic coverage (18,000+ genes), eliminates the need for *a priori* target selection, a requirement during hypothesis generation, immune atlas construction, or transcriptional niche discovery across whole-tumor landscapes. This benefit accompanies a structural limitation. Visium HD bins at 8 μm resolution capture transcripts from 2–4 cells per bin, rising to 5–10 cells at 16 μm aggregation, meaning cell-type-specific spatial signals require computational deconvolution rather than direct cellular measurement (65). Such resolution suffices and often proves statistically advantageous for questions concerning spatial gene program gradients, bulk immune infiltration architecture, or whole-section neighborhood ecology. However, it proves analytically inadequate when resolving intercellular distances, subcellular compartmentalization, or rare immune subsets that demand single-cell precision. FFPE compatibility via CytAssist workflow, combined with lower per-sample costs (~$500–1,500 USD for library preparation), renders sequencing-based platforms practical for biobanked discovery cohorts. Downstream computational demands remain substantial (35, 75).

Imaging-based *in situ* platforms (Xenium, CosMx, MERSCOPE) become the analytically superior choice during the transition from discovery to confirmation, when prior data defines candidate spatial biomarkers (CD8^+^ T-cell exclusion distances, TLS-associated gene programs, tumor-leading-edge immune exclusion signatures) requiring subcellular spatial fidelity. Platform differentiation rests not on panel size but signal integrity. Systematic benchmarking across six FFPE human tumor types showed Xenium exhibited a negative probe signal rate of 0.06%, yielding a gene-to-noise count ratio of approximately 450:1, versus CosMx’s 10.31% negative probe rate and 3:1 ratio, a >100-fold background difference consequential for low-abundance immune regulatory transcripts such as FOXP3, TIGIT, and LAG3 (65). Xenium accordingly showed stronger correlation with sequencing-based ground truth (Pearson R = 0.93 vs. R = 0.86 for CosMx versus Visium v2 reference), better spatial autocorrelation of tumor-contextual genes, and higher fidelity for endothelial and immune cell localization (65). These metrics yield direct biological consequences. In one multi-cancer benchmark, CosMx misassigned 71.6% of stromal fibroblasts as cancer cells at the tumor-stroma interface versus 10.2% for Xenium in matched colorectal sections, profoundly impacting immune exclusion scoring (65). Independent FFPE lung adenocarcinoma and pleural mesothelioma TMA benchmarking confirmed Xenium (unimodal and multimodal segmentation combined) achieved a false discovery rate below 0.09%, versus 5.8–10% for CosMx and 4.4–6% for MERSCOPE (76). Platform selection should prioritize the abundance of target transcripts, FFPE tissue quality (DV200), and the biological precision required. Xenium offers superior specificity and FFPE robustness, while MERSCOPE provides open-panel customization and simultaneous RNA–protein profiling on the same slide when functional protein readouts prove essential without separate experiments (76, 77). Higher costs (~$2,000–4,000 USD per run) and panel inflexibility render imaging platforms unsuitable for exploratory work, reinforcing staged workflow rationale.

Multiplexed spatial proteomics (IMC, MIBI, CODEX) warrants selection precisely when biological mechanisms operate at the protein level and resist reliable mRNA inference. This constraint rests on quantitative grounds. Same-section spatial co-profiling across five FFPE tumor types yielded a mean Pearson correlation between transcript and protein abundance of approximately 0.1, versus a bulk-level average of 0.7 across seven cancer types in Clinical Proteomic Tumor Analysis Consortium (CPTAC) datasets (65). In practical terms, this near-zero spatial correlation means that knowing a gene is highly expressed in a tissue region tells us very little about whether its corresponding protein is actually present and active there — a critical distinction when studying immune checkpoint ligands or signaling molecules whose function depends on protein-level activity rather than transcript abundance. Spatial RNA–protein decoupling reflects post-transcriptional regulation, protein turnover, localized secretion, and antibody-accessible epitope exposure invisible to transcriptomics. Consequently, immune checkpoint ligand distributions (PD-L1 spatial gradients), phosphorylation-state-dependent macrophage polarization, and spatially heterogeneous CAF activation evade reliable mRNA proxy capture at single-cell or regional resolution (78). Spatial proteomics platforms resolve functional states directly via panels of 40–100 validated markers, distinguishing activated signaling architectures within tumor niches. Operational constraints, 1–4 slides per day throughput, extensive antibody validation, per-slide costs of ~$1,500–3,000 USD, confine these platforms to mechanistic hypothesis testing on prioritized samples rather than cohort-scale profiling.

Platform properties converge into translational logic mapping research maturity to measurement modality. During discovery, when questions concern spatial architectures, cell-state neighborhoods, or gene programs distinguishing immune-evasive from immune-infiltrated tumor microenvironments, sequencing-based spatial transcriptomics (Visium HD or Stereo-seq) supplies unbiased whole-section landscapes for hypothesis generation, accepting multi-cell resolution trade-off for transcriptome-wide coverage (79). During validation, hypotheses are reduced to defined candidate spatial biomarkers that require cellular or subcellular confirmation via imaging-based platforms (Xenium or CosMx) delivering resolution and signal fidelity for quantitative precision. Critically, validation panels should avoid recapitulating discovery breadth. The Xenium 5K panel detected ~3-fold fewer transcripts per gene than the 377-gene panel on matched tissue, dropping MYC-positive cell classification from 70% to 30% and illustrating maximal panel size compromises per-gene sensitivity and risks biologically misleading cell-state estimates (65). During clinical translation, where cost, turnaround, and archival compatibility constrain deployment, targeted protein panels or computational spatial inference tools (Path2Space, PFMSP) extend validated biomarkers to routine specimens.

The paramount analytical error in spatial immunology treats platform selection as mutually exclusive. Observed spatial RNA–protein Pearson correlation of ~0.1, five to seven times below bulk equivalent positions transcriptomics and proteomics as orthogonal observations across regulatory strata (65). Transcriptomic profiling identifies CD8^+^ T-cell neighborhood phenotypic state in immune-excluded regions; corresponding proteomic profiling determines functional activation, protein-level exhaustion, or PD-L1^+^ suppression by proximate cells, information unbridgeable by Spearman correlations of 0.3–0.6 between mRNA and protein for most immune regulatory genes (73). A melanoma proof-of-concept combined mRNA–spatial protein model (AUC 0.97) outperformed transcriptomic (AUC 0.93) or spatial proteomics models alone (AUC 0.87), with DSP data clustering discretely from bulk RNA and affirming non-overlapping information (73). This orthogonality principle was operationalized further in a 2025 hepatocellular carcinoma study, where a graph neural network incorporating spatial multi-omic input achieved ROC-AUC > 0.90, predicting immunotherapy response, unapproachable by unimodal approaches, with predictive features including interface niches expressing restrictive extracellular matrix physically separating tumor from lymphoid aggregates in non-responders.

### 2.4 Multimodal data fusion and cross-modal integration

Spatially resolved studies increasingly demand integrative frameworks that can harmonize transcriptomic, proteomic, and histo-morphological modalities within a shared spatial coordinate system. When acquired across serial tissue sections, accurate registration using morphological landmarks or deep learning based alignment algorithms ensures physiological correspondence between molecular features and anatomical boundaries (80–83). Normalization across modalities must be correct for confounders such as sequencing depth, fluorophore intensity variability, and fixation artifacts. Comparative analyses indicate that imaging-based transcriptomics platforms such as CosMx and Xenium exhibit relatively stable count distributions across sections, though cross-platform variability remains substantial.

Data integration strategies broadly fall into early, late, and hybrid fusion. Early fusion concatenates normalized molecular and histological features at the single-spot or single-cell level before downstream modeling. Late fusion combines independent modality-specific models, whereas hybrid fusion employs embedding-based deep-learning frameworks and attention mechanisms that dynamically weigh the contribution of each modality depending on spatial context (84). In a 2025 study focused on hepatocellular carcinoma, a graph neural network using multimodal spatial transcriptomic–proteomic input predicted immune checkpoint inhibitor response with an ROC-AUC exceeding 0.90 and identified an extracellular matrix-enriched peritumoral region as a dominant barrier in non-responders (85). The incorporation of histopathology-derived embeddings from H&E and immunofluorescence images further enhanced model interpretability and improved prediction accuracy for immune infiltration gradients and stromal barrier strength. These developments mark a shift toward computationally unified spatial modeling, where AI-driven frameworks capture the emergent organization of tumor–immune interfaces beyond simple molecular annotation.

### 2.5 AI-based spatial modeling and predictive multi-omic networks

AI has become a critical engine for decoding the multidimensional complexity of spatial omics. By integrating transcriptomic, proteomic, metabolomic, and histological features within spatial coordinates, AI-driven platforms now surpass traditional bioinformatics pipelines in resolving tissue heterogeneity and cellular interaction networks (16, 86). The computational frameworks enabling this integration, including graph neural networks, vision transformers, variational autoencoders, and hybrid architectures, are examined in detail in Section 4, where their specific contributions to decoding immune evasion topology are discussed. Here, we focus on the multimodal alignment and fusion strategies that prepare spatial data for downstream AI modeling.

## 3 The architecture of evasion: spatial phenotypes of immunotherapy resistance

We stratify spatial findings by evidence level, computational (AI-derived associations and statistical correlations), experimental (perturbation studies and orthogonal assays), and clinical (prospective trials and multi-institutional cohorts), applying these designations consistently throughout. Spatial AI identifies candidate patterns; causal mechanisms require experimental and clinical validation. Immunotherapy resistance manifests as topological failure in tumor microenvironment (TME) architecture, not merely cell-intrinsic states. Stromal barriers, immune niche organization, tertiary lymphoid structure (TLS) maturation, and suppressive cell neighborhoods spatially dictate effector engagement. This section synthesizes four canonical evasion phenotypes, including immune exclusion, desert-inflamed continuum, TLS organization, and immunosuppressive neighborhoods, prioritizing multi-omics spatial data and topological metrics (distances, path lengths, adjacency, clustering coefficients) over single-cell molecular signatures alone. Emerging evidence indicates that resistance to immune-checkpoint blockade is not simply a cell-intrinsic phenomenon, but a topological one driven by the spatial architecture of the TME. The physical arrangement of stromal barriers, immune niches, tertiary lymphoid structures, and suppressive cell neighborhoods defines whether anti-tumor immunity can engage effectively. In this chapter, we review four major spatial phenotypes implicated in immunotherapy failure, including immune exclusion at the tumor–stroma interface; the desert-inflamed continuum of immune infiltration; tertiary lymphoid structure (TLS) formation and maturation; and spatially organized immunosuppressive cell neighborhoods. For each, we emphasize recent multi-modal spatial data and focus on topological metrics, distances, neighborhood structure, and adjacency rather than only single‐cell molecular signatures.

### 3.1 Immune exclusion and the tumor–stroma interface

A recurring feature of immunotherapy resistance is the immune exclusion of cytotoxic T cells from tumor nests, mediated by specialized cancer-associated fibroblast (CAF) subsets and an extracellular matrix (ECM) architecture. Together, these components constitute what we term the CAF-driven stromal barrier, a physical and molecular boundary composed of CAF populations and remodeled ECM that prevents immune infiltration. Hereafter, we use ‘stromal barrier’ as the primary term for this structure. In one landmark study of human non-small-cell lung carcinoma (NSCLC), researchers used single-cell RNA-sequencing coupled with multiplex immunohistochemistry and spatial imaging to identify two CAF populations strongly associated with T cell exclusion: MYH11^+^ αSMA^+^ CAF, which form a single‐cell layer lining cancer aggregates, and FAP^+^ αSMA^+^ CAF that assemble in patches or multiple layers around tumor nests. Although prospective validation is pending, the spatial exclusion phenotype correlates with poor outcomes in retrospective analyses (computational association; retrospective correlation). CAF–ECM programs colocalize with T-cell exclusion, suggesting biophysical/chemokine barriers, though causality requires experimental perturbation (hypothesis). Analogous phenotypes appear across cancers: HNSCC spatial transcriptomics reveal galectin-9^+^/CXCL9-12^+^ CAF subtypes enriched in immune-excluded stroma (computational association), while NSCLC identifies MYH11^+^/FAP^+^ CAF layers forming collagen XI/XII barriers (experimental validation via IHC/spatial proteomics). These manifest as “stromal barrier” node clusters increasing effector–tumor path length, reducing immune connectivity, a topological metric superior to bulk T-cell density for predicting ICB resistance. Mechanistically, these CAF–ECM programs generate biophysical and chemokine barriers to T-cell entry. Although direct immunotherapy response data were limited in that study, the spatial exclusion phenotype correlates with poorer outcomes and suggests that targeting these stromal architectures may enhance ICB efficacy.

Across other tumor types, analogous barrier phenotypes have been observed. CAF subtypes defined via spatial transcriptomics in head and neck squamous cell carcinoma (HNSCC) show enrichment of galectin-9 and CXCL9/10/12, co-localizing with immune-excluded stromal zones, though the precise topological metrics remain to be fully quantified. The key insight is that CAF heterogeneity is spatially organized, and immune infiltration is regionally suppressed where dense CAF/ECM architecture dominates. From a graph-based modeling perspective, these regions can be represented as “stromal barrier” node clusters that increase path-lengths between effector T-cells and tumor nodes, thereby reducing effective immune connectivity.

### 3.2 Immune desert versus inflamed phenotypes: a spatial continuum

The simplistic classification of tumors into desert, excluded, and inflamed understates the complex spatial architecture of immune contexts. Spatial-omics data now define a continuum of infiltration and suppression, where even “inflamed” tumors may harbor local micro-architectural pockets of dysfunction.

For instance, a spatial transcriptomic study in NSCLC treated with anti-PD-1/PD-L1 therapy showed that enrichment of CD163^+^ tumor-associated macrophages (TAMs) within intratumoral regions was associated with worse outcome, despite global immune infiltration, suggesting that neighborhood composition is crucial (87). Another study in esophageal squamous cell carcinoma (ESCC) found that the baseline distance of PD-1^+^ T-cells (and dendritic cells, macrophages) from tumor cell clusters predicted overall survival under chemoradiotherapy plus PD-1 blockade, and shorter distances indicated better outcome (88). Spatial transcriptomics in anti-PD-1/PD-L1-treated NSCLC reveals intratumoral CD163^+^ TAM enrichment predicting worse outcomes despite global infiltration (computational association; retrospective cohort), suggesting neighborhood composition determines resistance, though whether TAMs actively suppress or mark pre-existing states requires experimental validation (87). In ESCC, PD-1^+^ T-cell/tumor distance under chemoradiotherapy+PD-1 blockade predicts survival (shorter distances better; computational association; retrospective survival) (88). Multiplexed ion-beam imaging in high-grade serous carcinoma shows B-cell/M1 macrophage clustering and immune–immune adjacency predicting PFS/OS (n=72; HR 0.58, p=0.003; computational association; independent cohort) (89). Synthesis: Immune cell presence alone fails; topological metrics (infiltration distance, clustering coefficient, network density) govern efficacy, pending experimental causality confirmation. Thus, the mere presence of immune cells in a tumor is insufficient; spatial metrics such as infiltration distance, clustering coefficient, and immune network density are critical. In modeling terms, one may define: (i) the minimum Euclidean distance between an effector T-cell node and the nearest tumor-cell node; (ii) the clustering coefficient of immune-cell neighborhoods around tumor nests; (iii) immune–immune adjacency density within tumor core regions. These metrics refine immune phenotyping beyond simple counts or densities.

### 3.3 Tertiary lymphoid structures: location, organization, and function

Tertiary lymphoid structures (TLSs) are ectopic immune aggregates that resemble secondary lymphoid organs, comprising B-cell zones, T-cell zones, germinal centers, and high‐endothelial venules (HEVs). Their presence, maturity, spatial orientation, and distance to tumor parenchyma influence immunotherapy response and prognosis. In pancreatic ductal adenocarcinoma (PDAC), neoadjuvant immunotherapy induced the formation of mature TLSs enriched in IgG^+^ plasma cells and HEV‐like endothelial phenotypes; These were associated with improved survival (experimentally validated; neoadjuvant cohort) (87). A pan-cancer spatial transcriptomic analysis across 23 tumor types identified perivascular CCL19^+^ “lymphoid organizer” cells and endothelial HEV phenotypes co-localized with TLSs and found that mature TLS signature correlated with better outcomes (computational association; pan-cancer retrospective) (90). In gastric cancer, TLSs located within the tumor core correlated with higher densities of CD8^+^ LAG-3^−^, PD-1^+^, TIM-3^−^ T cells, and improved immune-related overall survival (computational association; retrospective cohort) (91). In HNSCC, the distance between TLS aggregates and tumor nests discriminated responder’s vs non-responders to checkpoint blockade (computational association; retrospective): responders had TLSs closer to tumor clusters and enriched B-cell peri-regions (92). Key topological features thus include (i) TLS center-to-tumor-cell cluster distance; (ii) internal TLS cell‐type density and inter-cellular proximity; (iii) maturity indicators (HEV presence, plasma-cell zones). Graphically, TLSs may be modeled as high-connectivity immune-subgraphs adjacent to tumor-cell nodes; numerical features (radius, node-density, adjacency to tumor nodes) can serve as explainable biomarkers.

### 3.4 Spatial mapping of immunosuppressive cell neighborhoods

Resistance is often driven by discretely localized immunosuppressive neighborhoods, where clusters of suppressive cell types (TAMs, regulatory T cells, immunosuppressive CAFs) are arranged in spatial architectures that impede effector immunity. A recent pan-cancer single-cell/snRNA atlas of TAM populations across nine tumor types found that pro-tumor TAMs preferentially locate in immune-excluded or hypoxic niches, whereas pro-inflammatory TAMs are found in immune-rich neighborhoods with exhausted CD8^+^ T-cells (computational association; pan-cancer atlas) ( (93). Spatial co-localization analyses showed suppressive TAM subsets occupy discrete domains where effector–tumor engagement is minimal. In HNSCC, the MHC-Ihi Gal9^+^ CAF subset forms stromal belts that locally trap effector T-cells and express inhibitory ligands *in situ* (computational association; spatial transcriptomics); whether these CAFs directly suppress T-cell function or reflect pre-existing exclusion requires experimental perturbation to confirm. In gastric cancer TLS landscaping, non-TLS regions exhibited increased intercellular distances and elevated local expressions of macrophage migration-inhibitory factor (MIF) and galectin signaling compared to TLS regions, correlating with suppressed immune-cell crosstalk (**Table 1**) (88). Spatial-AI models represent these suppressive neighborhoods as node clusters of high centrality, low effector adjacency, and long path‐lengths to tumor nodes, quantifying architectural immune paralysis.

**Table 1:** Quantitative spatial features of immune evasion.

Spatial feature	Biological meaning	Cancer type(s)	Technology	Immunotherapy relevance	References
Distance of effector T cells to tumor nests	Shorter distances indicate effective infiltration; longer distances reflect immune exclusion	ESCC, NSCLC, HNSCC	10x Visium ST, multiplex IHC	Short baseline distances predict better PD-1 blockade response	(94, 95)
Tumor-core vs stroma immune-cell density ratio	Low ratio denotes an “immune desert”; high ratio indicates active infiltration	Gastric cancer, PDAC	MIBI-TOF, spatial transcriptomics	Core-enriched TLS correlates with survival	(90, 96)
CAF barrier/ECM alignment	Physical/molecular barrier excluding T cells	NSCLC, HNSCC, HCC	scRNA + spatial proteomics, SHG ECM	Dense FAP^+^/αSMA^+^ CAF regions associate with non-response	(88, 89, 97)
Neighborhood composition/adjacency	Suppressive vs effector cell clustering	Pan-cancer, OC, GC	MIBI, CODEX, snRNA + imaging	High effector–effector adjacency supports response; suppressive clusters indicate resistance	(98, 99)
TLS–tumor distance	Proximity of TLS to tumor nests	HNSCC, GC, PDAC	ST, mIHC, RNAscope	Closer TLSs are associated with clinical responders	(91, 92, 100)
TLS maturity index	HEV presence and germinal center formation reflect functional competence	PDAC, multi-cancer	ST, imaging mass cytometry	Mature TLSs enhance antigen presentation and response	(92, 96)
Inter-cellular proximity within TLSs	Spatial intimacy between immune subsets facilitates interaction	GC, HNSCC	ST, MIBI	Reduced spacing corresponds to higher immune activation and survival	(100)
Suppressive neighborhood density	TAM + Treg + CAF cluster density	Pan-cancer, HNSCC, GC	snRNA-seq + spatial mapping	High suppressive density predicts resistance; effector-adjacent TAMs link with response	(93, 99)
Localized inhibitory-ligand enrichment	Gal-9, MIF, CSF1R clustering	HNSCC, GC	Multiplex IHC, spatial proteomics	Focal ligand enrichment indicates immunotherapy non-response	(93)
Graph-based connectivity metrics	Path length, clustering, modularity	Cross-cancer	GNNs representations	Low path length is associated with response; high modularity indicates resistance	(18, 101, 102)

### 3.5 Quantitative spatial metrics and topological motifs

Quantitative spatial descriptors derived from high-resolution imaging and spatial-omics data increasingly serve as biomarkers of immunotherapy response and resistance. These features capture the geometric and functional organization of immune–tumor ecosystems.

Distance to tumor boundary or tumor-cell clusters: The Euclidean separation between effector immune nodes (CD8^+^ T cells, TLS centers) and tumor cells defines immune exclusion. Larger distances indicate stromal or CAF-mediated barriers, while shorter distances predict effective infiltration and favorable PD-1 blockade outcomes (**Table 1**) (94, 95).

Core-to-stroma immune-cell density ratio: The ratio of immune-cell densities within tumor nests to those in stroma quantifies infiltration efficiency. Low ratios mark “immune-desert” states, whereas high intratumoral densities correlate with cytotoxic and B-cell enrichment and improved checkpoint blockade response (97).

Neighborhood composition and adjacency: Local adjacency between suppressive (TAMs, Tregs, CAFs) and effector (CD8^+^, NK) cells shapes the immune tone. In NSCLC, close suppressor–effector contact marks non-responders, while effector clustering predicts durable benefit (98, 99).

TLS maturity and organization: TLSs progress from immature aggregates to mature HEV^+^, BCL6^+^, IgG^+^ plasma-cell structures supporting antigen presentation. Greater maturity consistently is associated with enhanced survival and immunotherapy benefit (96, 100).

TLS maturity and organization: Reduced mean distances among B, Tfh, and dendritic cells within TLSs enable efficient immune crosstalk, corresponding to higher activation signatures and better outcomes in head-and-neck cancer (100).

Localized suppressive-signal enrichment: Spatial clustering of inhibitory ligands such as galectin-9, MIF, and CSF1R at effector–tumor interfaces indicates focal immunosuppression and resistance (96).

Graph representation of the TME: In graph models, cells or micro-regions are nodes, and spatial adjacencies (< X µm) are edges. Features such as effector–tumor path length, clustering coefficients, suppressive-node centrality, and graph modularity quantify spatial coordination. Deep learning, particularly graph neural networks (GNNs), integrates these topological metrics to generate explainable biomarkers of resistance and response (19, 103, 104).

## 4 Spatial AI: deep learning architectures for topological analysis

**Figure 2:**
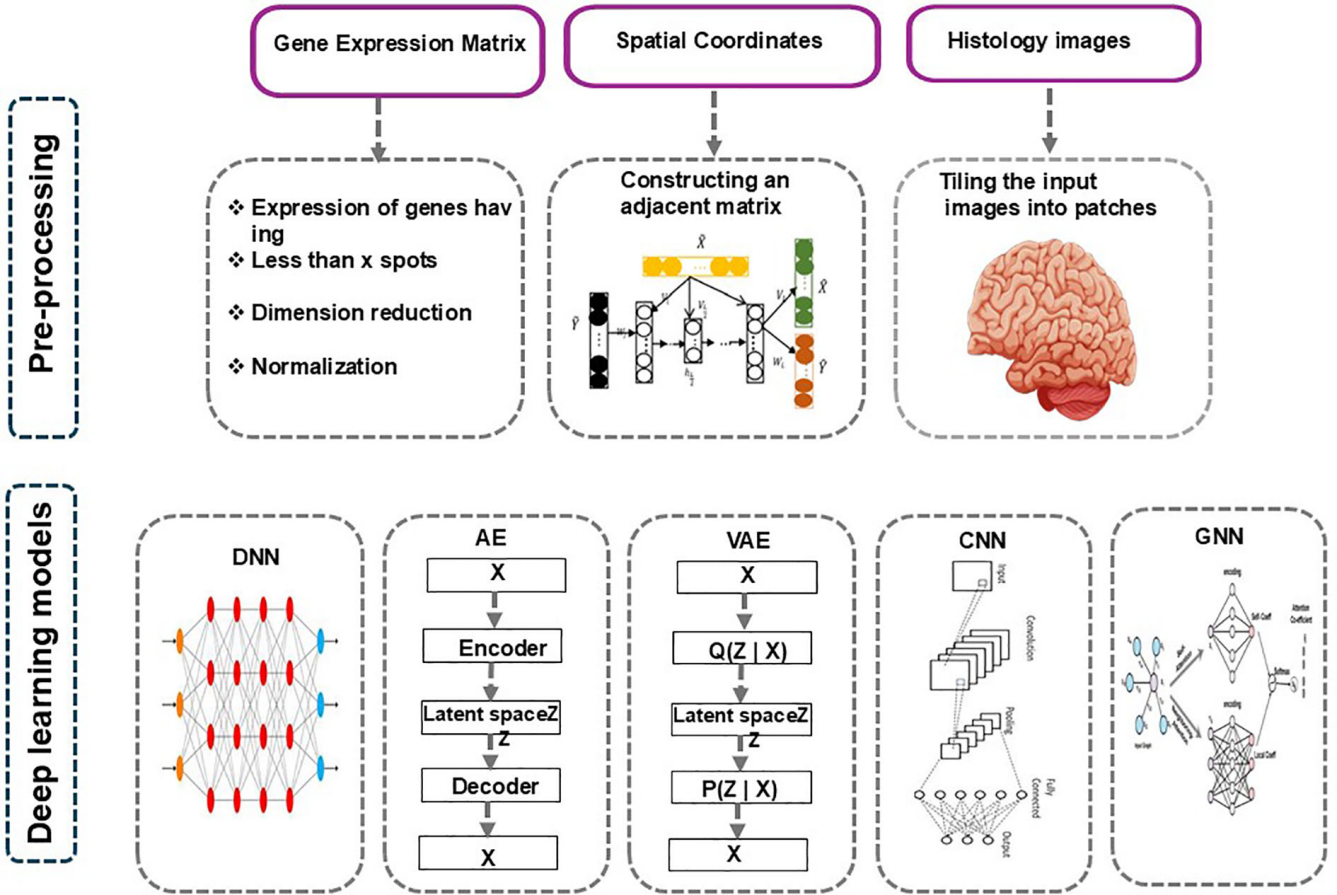
Overview of preprocessing and deep learning models for spatial omics analysis.

Several deep learning and hybrid AI frameworks have been proposed to address the inherent complexity of high-dimensional multi-omics datasets, as summarized in **Figure 2**. The process begins with pre-processing, where gene expression matrices are filtered, normalized, and dimensionally reduced; spatial coordinates are used to construct an adjacency matrix; and histology images are tiled into smaller patches. These pre-processed inputs feed into various deep learning models, including traditional deep neural networks (DNNs), autoencoders (AE), variational autoencoders (VAE), convolutional neural networks (CNNs), and graph neural networks (GNNs). Each model leverages different data modalities, gene expression, spatial structure, or tissue morphology, to learn latent representations that support downstream biological insights such as cell–cell interactions, tissue architecture, and disease-associated patterns (**Figure 2**). DeepProg integrates deep learning and traditional ML models to predict patient survival subtypes across multiple cancers (**Table 2**) (105). As shown in **Figure 2**, DNNs and CNNs process gene expression matrices and histology image patches respectively to extract local spatial features, while AEs and VAEs learn compressed representations of tissue structure useful for denoising and unsupervised domain discovery; GNNs operate on the adjacency matrix derived from spatial coordinates to model cell–cell relationships and identify interaction-driven patterns of immune evasion. OmiEmbed, a unified multi-task deep learning framework, allows tumor-type classification, clinical feature reconstruction, and survival prediction (105). Similarly, MetaCancer applies deep neural networks for pan-cancer metastasis prediction using multi-omics data (107), while DeepDRK uses a kernel-based deep learning framework to predict drug responses in cancer cells (108). Graph-based methods such as MoGCN (109) and PiLSL (110) exploit graph convolutional and neural networks for cancer subtype analysis and synthetic lethality prediction, respectively.

**Table 2:** Artificial intelligence-based omics data integration computational tools.

Name	Methods	Function	Reference
DeepProg	Deep learning + ML ensemble	Prediction of patient survival subtypes (across cancers)	(105)
127OmiEmbed	Deep learning (multi-task)	Tumor-type classification, embedding, survival prediction (multi-omics)	(106)
MetaCancer	Deep neural network	Pan-cancer metastasis prediction (multi-omics)	(107)
DeepDRK	Deep learning kernel-based	Cancer cell line/patient drug-response prediction (multi-omics)	(108)
MoGCN	Graph convolutional network	Cancer subtype classification via multi-omics GCN	(109)
PiLSL	Graph neural network	Synthetic lethality prediction in cancer using multi-omics data	(110)
MANAclust	Machine learning/clustering	Identification of disease endotypes in cancer (multi-omics + clinical)	(111)
PathME	Deep learning/pathway‐based autoencoder	Patient clustering in cancer, pathway-level multi-omics integration	(112)
SCA	Deep learning autoencoder	Extract hidden biologically relevant features from cancer single-cell or bulk multi-omics	(113)
DeFusion	Machine learning/denoising network regularization	Noise reduction & integration of cancer multi-omics data	(114)
netDx	Machine learning/patient-similarity networks	Interpretable patient classification across cancer subtypes	(115)
wMKL	Weight-boosted multi-kernel learning	Novel cancer subtype identification	(116)
SNRMPACDC	Deep learning + GCN	Anticancer synergistic drug combination prediction	(117)
GraphCDR	Graph neural network + contrastive learning	Cancer drug response prediction using multi-omics integration	(118)
DeepKEGG	Deep learning interpretable framework	Cancer recurrence prediction & biomarker discovery (multi-omics)	(119)

To address cancer heterogeneity, unsupervised models like MANAclust (111) and PathME (112) facilitate patient stratification and pathway-level clustering, while SCA (113) leverages sparsely connected autoencoders for extracting biologically meaningful features from single-cell omics. For denoising and data regularization, DeFusion provides a robust ML-based framework for integrating noisy multi-omics profiles (114). netDx (115) employs interpretable patient-similarity networks to classify cancer patients, and wMKL (116) enhances cancer subtype identification using weight-boosted multi-kernel learning. Moreover, SNRMPACDC (117) and GraphCDR (118) apply deep learning and graph contrastive frameworks to predict anticancer drug synergy and drug response, respectively. Finally, DeepKEGG (119) integrates biological pathway information for recurrence prediction and biomarker discovery in cancer. Collectively, these AI-based computational approaches demonstrate how multi-omics integration can reveal clinically actionable insights and accelerate precision oncology.

### 4.1 Statistical learning and architecture classes in spatial biology

Before exploring specific applications, it is important to define the computational foundations of Spatial AI, since different model classes address distinct facets of the spatial inverse problem. In spatial omics, AI and machine learning learn patterns directly from data, allowing them to fill in biological details that are missing or obscured by technical noise, an essential capability when the spatial organization of tissues is too complex to model with simple statistical rules. Deep learning extends this principle by using multi-layer nonlinear models that automatically learn hierarchical spatial features without manual engineering, an essential property when relevant patterns are unknown or difficult to formalize. Each neural architecture encodes different geometric assumptions suited to particular data types. When the primary data source is a tissue image or a spatially binned gene expression map arranged on a regular grid, a different computational approach is needed than when the data represents irregular networks of interacting cells. Convolutional neural networks (CNNs) operate on Euclidean grids, making them effective for histology and spatially binned transcriptomic data; their translational invariance allows detection of meaningful spatial textures and organization across scales (120). However, biological tissues are not grids — cells are irregularly positioned and communicate through a web of physical contacts and signaling interactions that CNNs cannot naturally represent. Graph neural networks (GNNs) generalize this approach to non-Euclidean tissue graphs, modeling relationships between cells or spatial spots via message passing along biologically meaningful edges that capture proximity and intercellular communication.

When the goal shifts from detecting patterns to understanding which specific cellular interactions are most important for a given outcome, models that can explain their own decisions become essential. Transformer architecture expands these ideas through attention mechanisms that dynamically weight spatial context, learning which regions or cells are most informative for a given task rather than relying on fixed receptive fields. Vision transformers process histological patches to capture long-range dependencies, while graph transformers build attention over cellular neighborhoods. Generative models, including autoencoders, variational autoencoders (VAEs), and generative adversarial networks (GANs), further extend the framework by learning latent representations of tissue structure. Autoencoders provide denoising and dimensionality reduction, VAEs impose probabilistic structure for uncertainty estimation, and GANs generate realistic tissue-like samples for data augmentation. The optimal architecture depends on the geometry of the inverse problem: CNNs and vision transformers are ideal for image-based inference, GNNs and graph transformers for interaction and niche discovery, hybrid CNN–GNNs models for multimodal fusion, and generative frameworks for counterfactual simulations. This architectural diversity reflects functional specialization, not redundancy, ensuring that computational models align with the geometric and biological structure of the underlying spatial system (103).

### 4.2 From pixels and spots to graphs: practical graph construction

Graph models provide a natural framework for representing tissue architecture, where nodes denote cells, nuclei, or spatial transcriptomic spots, and edges represent physical or functional interactions. The construction of these graphs critically shapes biological interpretation. In single-cell or multiplexed imaging data, each segmented cell serves as a node with multi-modal features such as RNA, protein, and morphology. For spot-based data (10x Visium), deconvolution methods like STdGCN infer pseudo-cell nodes from mixed signals to better capture spatial context (19, 104).

Edges are often defined using radius or k-nearest neighbor (kNN) rules within 10–30 μm, modeling short-range cell–cell communication (121). More advanced strategies incorporate ligand receptor probabilities, ECM barriers, or adaptive attention-weighted adjacencies that capture biologically relevant connectivity (122, 123). Edge attributes such as distance or signaling likelihood can further refine heterogeneity between node types.

Graph design must match the biological hypothesis. For instance, when analyzing immune exclusion, edges crossing stromal barrier zones should be annotated or down-weighted to reflect spatial constraints. Thus, biologically informed graph construction is foundational for interpretable graph neural network (GNNs) analysis of tissue topology.

### 4.3 Core GNN families and their role in spatial omics

Graph neural networks (GNNs) represent tissue architecture as a graph in which nodes encode individual cells or spatial spots and edges encode physical proximity or inferred signaling relationships. Rather than treating GNN variants as interchangeable options, each major family addresses a distinct mechanistic question that conventional bulk or single-cell approaches could not resolve: which spatial domains harbor immune resistance? which cell interactions drive suppression? and how can those topological signals be translated into validated clinical biomarkers? The following synthesis organizes GNNs families around these three biological challenges, highlighting what each uniquely contributes to the mechanistic understanding of immune evasion. The first challenge, delineating immunosuppressive spatial domains, is addressed by GCNs. SpaGCN aggregates normalized molecular features from spatial neighbors to produce context-aware embeddings that separate immune-excluded stromal margins from tumor cores, a resolution that bulk deconvolution cannot achieve (122). This spatial separation is a resolution that bulk deconvolution cannot achieve, as averaging signals across whole tissue sections obscures the boundaries between immunosuppressive and immune-active zones. STAGATE extends this by learning spatially adaptive adjacency weights within an attention-autoencoder, sharpening the boundary between immune desert and inflamed zones at single-spot precision (19). By precisely mapping where suppressive niches begin and end, these models generate spatial coordinates that can guide locoregional interventions, for instance, targeting CAF-dense exclusion zones without disrupting adjacent immune-active stroma.

The second challenge, decoding which interactions within those domains drive immune paralysis, is met by graph attention networks (GATs) and variational graph autoencoders (VGAEs). GAT attention coefficients are not merely technical parameters; they map which cell adjacencies most predict immunosuppressive outcomes, directly implicating specific ligand–receptor axes, such as those between PD-L1^+^ tumor cells and exhausted CD8^+^ T cells, or between CAF subtypes and excluded immune populations, and converting black-box predictions into spatially located, experimentally testable hypotheses (124). In doing so, GATs convert black-box predictions into spatially located, experimentally testable hypotheses about which cellular interactions drive resistance. VGAEs complement this by reconstructing suppressive sub-graphs, such as clustered TAM–Treg–CAF neighborhoods, from incomplete molecular profiles without requiring labelled outcome data, and by enabling in silico perturbation of niche composition to predict how topological changes, reduced Treg density, altered macrophage polarization, propagate through immune connectivity (125). Crucially, VGAEs also enable in silico perturbation of niche composition, allowing researchers to predict how topological changes such as reduced Treg density or altered macrophage polarization would propagate through immune connectivity before testing these hypotheses experimentally.

The third challenge, ensuring that mechanistic insight is reproducible at the scale required for clinical validation, is addressed by inductive and task-specific architecture. GraphSAGE trains on sampled local neighborhoods rather than full tissue graphs, enabling GNNs inference across whole-slide images containing millions of cells without prohibitive memory costs (126). STdGCN combines GCN-based spatial encoding with single-cell reference deconvolution to achieve cell-type resolved profiling of FFPE specimens at trial-relevant scale (127). These advances are not ends in themselves; they permit topological biomarkers discovered in small cohorts, effector–tumor path length, suppressive niche clustering coefficients, TLS connectivity indices, to be quantified reproducibly across the multi-institutional datasets required for regulatory validation. Together, GCNs and attention autoencoders define where suppressive architecture resides; GATs and VGAEs decode how cell interactions sustain it; and inductive frameworks determine whether these spatial resistance signatures can be reliably measured in clinical practice. **Table 3** presents a comparative analysis of deep learning architectures for spatial omics, detailing their advantages, limitations, and typical use cases.

**Table 3:** Comparative analysis of deep learning architectures for spatial omics.

Architecture	Strengths	Limitations	Best Use	Compute	Examples
CNNs	Efficient for images; strong local features; pretrained models	No native graph modeling; limited long-range context	Histology analysis; spatial gene prediction	Mod. (16–32 GB)	ResNet, U-Net
GNNs	Models cell–cell relations; flexible topology	Graph construction; scalability; over-smoothing	Cell interactions: spatial niches	High (32–80 GB)	GCN, SpaGCN
GATs	Attention-based interpretability; adaptive neighborhoods	High cost; attention instability	Explainable spatial inference	Very High (40–80 GB)	GAT, STAGATE
VAEs	Probabilistic latent space; uncertainty	Lower fidelity; tuning sensitive	Denoising; unsupervised clustering	Mod. (16–32 GB)	VGAE, stKeep
GANs	High-fidelity synthesis; conditional generation	Training instability; no uncertainty	Data augmentation; TME simulation	High (32–64 GB)	Graph-GAN
ViTs	Global context, strong transfer learning	Data-hungry; expensive	WSI analysis; multimodal fusion	Very High (≥80 GB)	ViT, Swin
Hybrid	Combines morphology + topology	Complex tuning; high compute	Multimodal spatial omics	Extreme (64–128 GB)	CNN-GNN

Convolutional Neural Networks (CNNs): CNNs efficiently model data arranged on regular grids, such as histology images and tiled spatial transcriptomics, by exploiting translational invariance and shared weights to enhance computational efficiency. Architectures like ResNet, DenseNet, and U-Net are often pre-trained on large datasets, capture fine textures and hierarchical tissue organization, with GPU-optimized implementations facilitating gigapixel-scale training. Their fixed receptive fields and grid constraints limit long-range spatial modeling and irregular graph processing. CNNs perform best in H&E image classification, segmentation, and morphological feature extraction, requiring moderate computational resources (16–32 GB GPU memory) (128).

Graph Neural Networks (GNNs): GNNs flexibly represent irregular spatial or cellular data by encoding intercellular relationships through graph edges based on proximity or ligand–receptor interactions. Their message-passing framework integrates biological priorities and captures emergent tissue properties but faces scalability challenges at single-cell resolution, sensitivity to graph definition, and potential over-smoothing in deep layers. Interpretability remains limited without attention mechanisms. GNNs excel in cell–cell interaction inference, spatial niche discovery, and molecular–spatial data integration, typically demanding high computational power (32–80 GB GPU memory) (129).

Graph Attention Networks (GATs) and Graph Transformers: Extending GNNs, these models learn adaptive attention weights that emphasize biologically relevant neighbors, improving interpretability and handling heterogeneous tissue contexts. Attention enables both local and global context modeling but increases computational cost and may yield unstable or correlational importance scores without regularization. They perform well in interpretable spatial biomarker discovery, therapy-response modeling, and multi-scale tissue analysis, yet require substantial GPU memory (40–80 GB) (124).

Variational Autoencoders (VAEs): VAEs learn probabilistic latent representations that quantify uncertainty and enable controlled generation of synthetic tissue architectures. Their regularized latent space supports smooth interpolation and integration of biological priors, making them well-suited for unsupervised learning with limited labels. Reconstructions can be blurrier than GAN outputs due to Gaussian assumptions, and latent interpretability often requires disentanglement constraints. VAEs are effective for dimensionality reduction, TME synthesis, and denoising, with moderate computational needs (16–32 GB GPU memory) (130).

Generative Adversarial Networks (GANs): GANs synthesize realistic tissue images by adversarially learning complex data distributions, excelling in data augmentation, histological simulation, and style transfer. However, training instability, mode collapse, and weak interpretability remain issues, and quality evaluation depends on expert assessment. They are particularly useful for generating histopathology of rare cancers, requiring considerable computational capacity (32–64 GB GPU memory) (131).

Vision Transformers (ViTs): ViTs use self-attention to capture long-range dependencies from the first layer, providing global context and interpretability through attention maps. Large-scale pre-training enables state-of-the-art performance across histology and multimodal analysis, though ViTs lack CNNs’ local inductive biases and require immense datasets and compute. Ideal for whole side classification and multimodal integration ViTs require substantial resources (> 80 GB of GPU memory or distributed systems) (132).

Hybrid Architectures (CNN + GNN, Transformer + GNNs): Hybrid models combine CNNs for local feature extraction, GNNs for spatial reasoning, and transformers for global context, enabling end-to-end integration of histology, transcriptomics, and proteomics. They provide flexible and interpretable multimodal modeling but demand extensive tuning, large datasets, and an additive computational cost. Most suitable for integrative spatial omics and clinical outcome prediction, they typically require high-memory or multi-GPU configurations (64–128 GB GPU memory) (133).

While each architecture class has distinct computational strengths, their value in spatial immune-oncology lies not in technical capability alone but in the biological questions they make answerable. CNNs reveal morphological correlates of immune exclusion directly from routine histology; GNNs and GATs expose the interaction networks that sustain suppressive niches and generate interpretable hypotheses about specific ligand–receptor axes driving resistance; VAEs and GANs enable controlled simulation of alternative TME states, allowing *in silico* testing of whether disrupting a suppressive neighborhood restores immune connectivity; and ViTs and hybrid architectures integrate signals across scales, capturing both local cellular interactions and global tissue organization that together determine whether cytotoxic T cells can physically reach tumor nests. Collectively, these architectures do not merely improve predictive accuracy; they provide a computational grammar for translating the spatial geometry of immune evasion into mechanistic understanding and, ultimately, into targetable vulnerabilities.

### 4.4 Self-supervised and contrastive approaches: pretraining for robustness

Spatial omics datasets are typically limited, heterogeneous, and noisy, motivating self-supervised learning (SSL) and contrastive pretraining to learn transferable graph embeddings before supervised fine-tuning. Mutual-information maximization methods such as Deep Graph Infomax (DGI) train node encoders by maximizing agreement between local patch representations and a global graph summary, producing node embeddings that are robust to noise and platform effects (124). Contrastive graph frameworks create augmented graph views and train embeddings to be invariant to such augmentations, which improves generalization and transfer across tissues and platforms (134). These generic graph SSL methods have been adapted for spatial omics: multimodal contrastive pipelines that align histology patches, spatial transcriptomic spots, and proteomic profiles improve domain discovery and cross-sample transfer; in practice, contrastive pretraining on large unlabeled tissue atlases yields embeddings that markedly boost downstream tasks such as spatial domain detection and therapy-response prediction (135).

### 4.5 Generative models and in-silico TME synthesis

Generative graph models such as variational graph autoencoders (VGAEs) and graph-GANs enable in silico simulation of tumor microenvironment (TME) topologies for data augmentation, hypothesis testing, and counterfactual analysis. VGAEs learn latent representations of cell–cell or spot–spot graphs derived from spatial transcriptomics or imaging data, then generate synthetic graphs conditioned on desired phenotypes. Increasing tumor-associated macrophage (TAM) density in a subregion while preserving overall structure. Such synthetic TMEs allow controlled perturbations, such as altering CAF adjacency or B-cell–TLS connectivity, to test how topological changes affect predicted therapeutic response. Graph-GAN frameworks extend this by adversarially generating both node features and edge patterns, creating realistic yet novel tissue graphs that mimic spatial heterogeneity. In spatial omics, GAN-based approaches like SST-editing perform in silico spatial transcriptomic editing at single-cell resolution, enabling gene-guided modification of tissue architecture (136). Similarly, heterogeneous graph learning frameworks such as stKeep integrate diverse node types and relational edges to reconstruct spatial networks and explore TME perturbations (137). Collectively, generative graph models provide a scalable route to simulate alternative TME states, evaluate spatial drivers of resistance, and design predictive in silico experiments that bridge discovery and therapeutic design.

### 4.6 Explainability and the limits of post-Hoc interpretation

Interpretability is essential for clinical deployment of Spatial AI, but a clear distinction must be made between intrinsic interpretability and *post-hoc* explanation. Much of current explainable AI (XAI) yields plausible associations rather than validated causal mechanisms, and conflating the two risks overstating the reliability of spatial biomarkers.

Intrinsic interpretability-building biology into models: The most robust path to interpretability is embedding biological knowledge directly into model architecture so that learned representations are mechanistically meaningful (138). In Spatial AI, this includes biologically constrained graph neural networks in which edges encode validated ligand–receptor interactions, diffusion-limited signaling ranges, or extracellular matrix barriers, allowing attention weights to reflect specific signaling axes rather than arbitrary feature combinations. Hybrid mechanistic models that couple neural networks with partial differential equations governing cytokine diffusion, metabolite gradients, or oxygen tension further enforce physical plausibility while learning unknown reaction terms (139). Explicit graph-theoretic features, such as immune–tumor distances, clustering of immunosuppressive niches, or tertiary lymphoid structure connectivity, yield interpretable scalar biomarkers, while identifiable latent variable models constrain representations to biologically grounded factors rather than opaque latent features.

*Post-Hoc* interpretability: *Post-hoc* explanation methods attempt to rationalize predictions from complex black-box models but face inherent constraints. Attention-based approaches highlight salient cells or regions yet capture correlation rather than causation and may be unstable across retraining. Subgraph explainers identify predictive cellular motifs and generate hypotheses, but do not establish causality without experimental perturbation. Counterfactual and feature-perturbation analyses assess model sensitivity to hypothetical changes, but their validity depends on the correctness of the underlying model and cannot distinguish causal drivers from correlated effects (140).

The stability and uniqueness problem: A key limitation of *post-hoc* XAI is the lack of stability and uniqueness of explanations. Different methods applied to the same model often yield conflicting importance rankings, and small changes in initialization or training data can substantially alter highlighted features, indicating that explanations may reflect arbitrary properties of learned representations rather than true biological mechanisms (140).

Path forward and clinical implications: *Post-hoc* explanations should therefore be framed as hypothesis-generating rather than validated knowledge. Spatial AI workflows must include stability testing across retraining and explanation methods, biological plausibility filtering, experimental validation using targeted spatial perturbations, and replication in independent cohorts. For clinical and regulatory use, intrinsically interpretable models grounded in biological constraints may require less extensive validation, whereas black-box models relying on *post-hoc* explainers demand rigorous sensitivity analyses, multi-institutional validation, and demonstrated explanation stability (140). Transparent acknowledgment of interpretability limits will strengthen confidence in Spatial AI and support responsible clinical translation.

### 4.7 Multiscale and multi-modal models: integrating WSI, ST, and proteomics

Spatial AI requires integration across scales, from subcellular structures to whole-tissue organization, and across multiple modalities, including histology, spatial transcriptomics, and proteomics. Contemporary pipelines adopt hybrid architectures where image patches are embedded via convolutional neural networks, molecular features are encoded with modality-specific encoders, and embeddings are fused either through concatenation or cross-modal attention to generate comprehensive node features for graph-based modeling (19). Such multimodal representations capture complementary biological information, enabling graph neural networks (GNNs) to learn topological and functional tissue organization more accurately. SPA-GNNs frameworks, which integrate SpaGCN with histological embeddings, and STAGATE combined with CNN-based image features, have demonstrated superior spatial domain identification and predictive performance relative to single-modality approaches (141). Moreover, cross-modal self-supervised learning, such as contrastive alignment between ST spots and corresponding histological patches, enhances feature coherence, reduces the requirement for large, labeled datasets, and improves generalizability across tissue types and experimental platforms (135).

### 4.8 Scalability and computational strategies for gigapixel data

**Figure 3:**
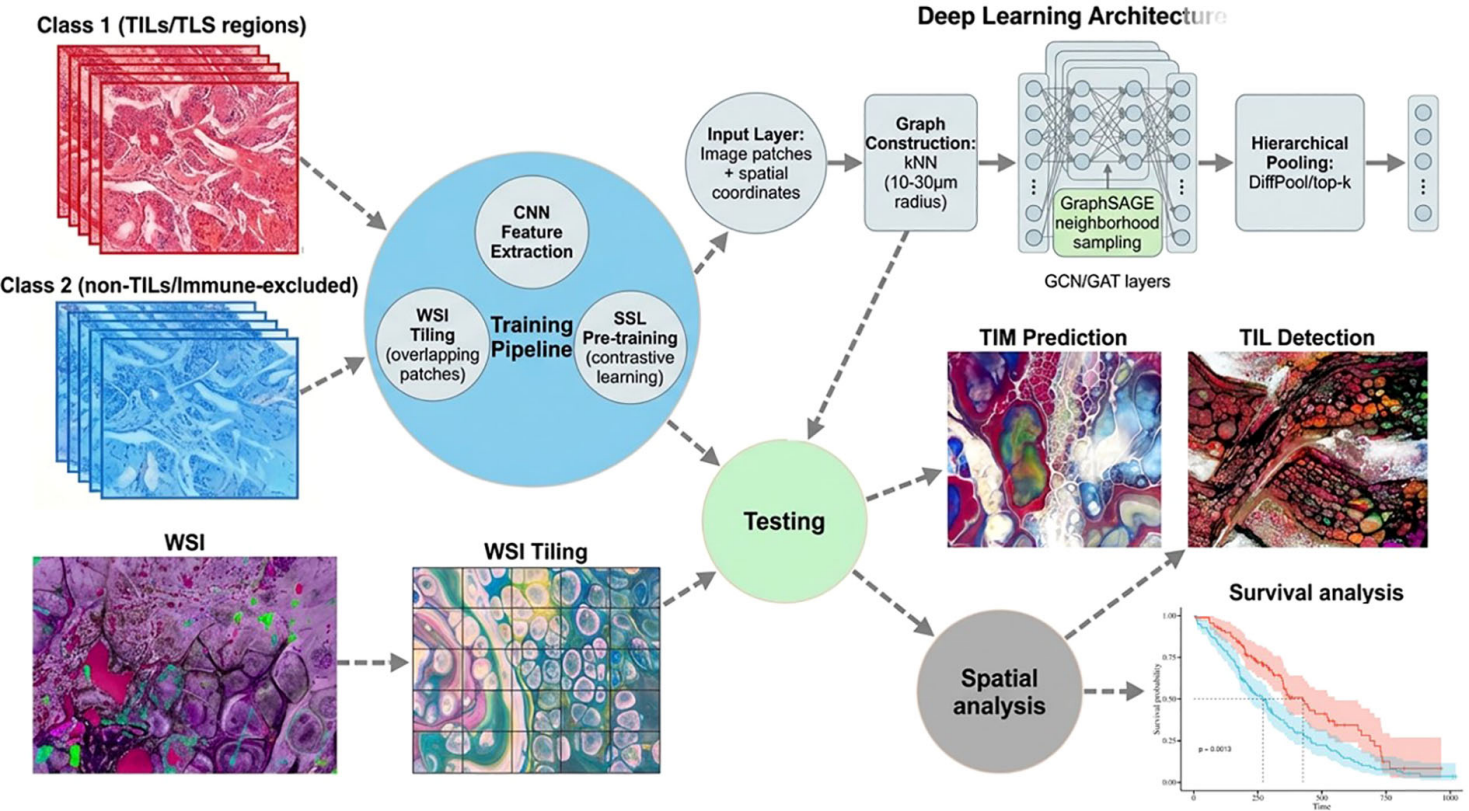
Deep learning pipeline for scalable spatial AI analysis of whole-slide images. The framework illustrates key computational stages: WSI tiling into overlapping patches, CNN-based feature extraction, graph construction using spatial coordinates and kNN connectivity, hierarchical pooling via GraphSAGE/GCN-GAT layers, and downstream outputs including tumor-immune microenvironment (TIME) prediction, TIL detection, and survival analysis. Tissue regions are classified into immune-active zones (Class 1: TIL/TLS-enriched) and immune-excluded zones (Class 2: non-TIL), with SSL pre-training via contrastive learning enhancing feature robustness across tissue types.

Processing whole-slide images (WSIs) at single-cell resolution generates extremely large graphs, often encompassing millions of nodes, which necessitate specialized computational strategies to enable feasible training and analysis (**Figure 3**). As illustrated in **Figure 3**, the framework classifies tissue regions into two functional categories: Class 1 regions containing tumor-infiltrating lymphocytes (TILs) and tertiary lymphoid structure (TLS) aggregates representing immune-active zones, and Class 2 regions representing non-TIL, immune-excluded areas — the spatial boundary between these classes being precisely the interface that Spatial AI aims to characterize and exploit therapeutically. One common approach is tiling, where the WSI is divided into overlapping subregions; each tile is processed independently, and graph embeddings are subsequently reconciled at tile boundaries using pooling and coarsening methods to preserve global tissue topology (142, 143). Hierarchical graph pooling techniques, such as DiffPool or top-k pooling, allow the model to learn multiscale representations by aggregating local neighborhoods into coarse-grained graph structures while retaining fine-scale spatial information for accurate local inference (144). For extensive graphs, out-of-memory training strategies and high-performance computing resources have become essential. Inductive GNNs models, including GraphSAGE, perform neighborhood sampling to reduce memory load, and distributed training across GPU or TPU clusters enables scalable processing of clinical-scale WSIs (145). Together, these computational strategies allow Spatial AI pipelines to efficiently model the full complexity of tissue architecture while maintaining interpretability and predictive power.

## 5 Therapeutic translation: targeting spatial signatures

Translating spatial signatures into therapy requires (i) robust predictive biomarkers that incorporate topological features, (ii) identification of spatially actionable targets and niches, and (iii) validated, explainable computational pipelines suitable for clinical deployment.

### 5.1 Predicting immunotherapy outcome using spatial signatures

Predictive models that incorporate spatial context outperform many bulk or single-marker assays since they capture the arrangement and neighborhood relationships of immune, stromal, and malignant elements that determine immune accessibility. High-dimensional multiplexed imaging and spatial transcriptomics studies have repeatedly shown that spatial organizations such as immune exclusion, the proximity of effector T cells to tumor nests, and immune-suppressive stromal pockets are associated with response or resistance to immune-checkpoint blockade (ICB) (146, 147).

A longitudinal, multiplexed imaging mass cytometry (IMC) study of breast cancer demonstrated that cell phenotype, activation state, and spatial location differ between ICB-sensitive and ICB-resistant tumors and that early on-treatment spatial changes predict therapeutic effect, indicating a strong association between spatial topology and clinical outcome (clinically validated; longitudinal IMC cohort) (146). Complementary work shows that deep learning applied to routine histology can predict PD-L1 status and other immune-related signatures, enabling surrogate spatial readouts from standard clinical specimens (147). Spatially informed gene signatures and spatial proximity metrics have been developed for melanoma and other cancers and improve response prediction when combined with molecular features (148).

Methodologically, graph-based representations where nodes are single cells or microregions and edges encode adjacency or inferred interaction allow explicit modeling of topological dependencies. Recent applications of GNNs to spatial omics yield enhanced prognostic models and extract interpretable subgraph features that relate to biological mechanisms of immune evasion (127). Together, these studies support that topological biomarkers, composite metrics that encode arrangement, neighborhood composition, and local interaction motifs, are powerful predictors of immunotherapy outcome.

### 5.2 Identifying actionable targets within the microenvironment

Spatial AI transcends “what to target” by answering “where to target.” Spatially resolved multi-omic profiling maps molecular programs to discrete micro-niches and thereby identifies location-specific vulnerabilities amenable to therapeutic intervention (149). Spatial proteogenomic analyses identify TGF-β and integrin signaling enriched within stromal barrier exclusion zones (computational association; spatial proteogenomics), suggesting these pathways as candidate targets for stromal-remodeling strategies, pending experimental validation (62).

Graph and attention-based deep-learning frameworks can prioritize candidate interventions by ranking ligand–receptor subnetworks and node-level features within high-risk topologies (150). These approaches permit coupling of spatially localized perturbations to predict restoration of immune cell infiltration and function, thereby converting descriptive maps into testable therapeutic hypotheses.

Causer et al. (151) used combined spatial transcriptomics and proteomics (SpiCi) in head-and-neck carcinoma to identify drug-resistant tumor edges and VEGFA immune-excluded zones, providing a rationale for stromal-remodeling interventions (151). Sharma et al. (152) implemented a transformer-based AI pipeline (SELFormer) with QSAR modeling to discover FDA-approved and natural compounds targeting TNFRSF10A/TRAILR1 in PDAC, integrating spatial transcriptomic data to link molecular targets with immune-excluded micro-niches (152). Paniagua et al. (155) introduced TG-ME, combining transformers and GraphVAE to dissect tumoral niches, revealing stage-specific niche compositions predictive of prognosis (**Table 4**) (155).

**Table 4:** AI and spatial omics models applied across cancer types.

Cancer type	Model/framework	Core methodology	Translational insight	Reference
CNS tumors	NePSTA (GNNs + ST)	Integrated histology + molecular diagnostics	Enhances CNS tumor classification from single sections.	(139)
HCC	SPARC	Spatial proteogenomic + GNN	Predicts ICB response (AUC > 0.9); identifies ECM-based resistance niches.	(153)
Ovarian	CNN + attention + ST	Links model attention to immune activation	Attention features highlight immune-active regions, improving prognosis prediction.	(129)
Breast, Lung	DL on WSIs	Spatial inference of mRNA/miRNA	Defines the heterogeneity index linked to survival.	(154)
OPSCC	SpiCi + ST	Spatial proteogenomics	Maps PD-1/PD-L1^low^ VEGFA^high^ niches; explains ICB failure.	(151)
PDAC	SELFormer + QSAR	AI-driven target/drug discovery	Identifies TRAILR1 modulators; spatially validated.	(152)
NSCLC	TG-ME(Transformer+ GraphVAE)	Integrates morphology + spatial transcriptomics	Dissects tumor niches and progression; guides personalized treatment.	(155)
Lung, Breast, CRC	GIST	Histology + Graph Transformer	Improves segmentation by ~50%; enhances marker discovery.	(156)
Breast	Path2Space	Deep learning inference of ST	Predicts > 4,300 genes; defines spatial subgroups with prognostic value.	(157)
Breast	PFMSP	Patch filter via ST	Predicts molecular subtype (80% accuracy); speeds diagnosis.	(158)
Multi-cancer	MISO	Multiscale integration of spTx + morphology	Predict gene expression + MSI from H&E slides.	(157)
Bladder, UTUC	ROICellTrack	Deep integration of IF + ST	Identifies cancer–immune mixtures; supports targeted therapy.	(159)
Ovarian HGSC	AI + ST	AI-guided ST profiling	Reveals outcome-specific subpopulations and chemoresistance pathways.	(160)
Multi-tissue	VORTEX	AI-driven 3D ST	Predicts volumetric 3D gene expression; non-destructive and scalable.	(161)
Prostate	CNN ensemble + ST	Morphology–gene correlation	Unsupervised mapping of molecular heterogeneity.	(162)
Liver (cHCC-CCA)	DL phenotyping + ST	Reclassifies biphenotypic tumors	Aligns predictions with genetics and outcomes.	(163)

### 5.3 Translational strategies and clinical implementation

**Multimodal integration and robustness:** Combining spatial transcriptomics, multiplex proteomics (IMC, MIBI), and histology improves signal fidelity and model generalizability. Frameworks that construct dynamic graphs across modalities reduce platform-specific noise and enhance detection of biologically relevant topologies (17).

Approaches such as GIST integrate histopathology foundation models with spatial transcriptomics via hybrid graph transformers, improving microenvironment segmentation by ~50% across cancers (156). MISO (157) and ROICellTrack (159) fuse H&E morphology, immunofluorescence, and spatial transcriptomics to extract spatially consistent multi-omic features (**Table 4****).** These models exemplify scalable, multi-modal pipelines essential for translational readiness.

In ovarian high-grade serous carcinoma, AI-guided spatial-transcriptomic profiling identifies outcome-specific subpopulations and chemoresistance pathways, enabling refined risk stratification (160). VORTEX, a cross-tissue 3D Spatial AI framework, predicts volumetric gene-expression landscapes in a nondestructive and scalable manner (161). In prostate cancer, CNN ensemble–ST integration enables unsupervised mapping of morphology–gene correlations, revealing spatial molecular heterogeneity (162). In combined hepatocellular–cholangiocarcinoma (cHCC-CCA), deep-learning phenotyping combined with spatial transcriptomics reclassifies ambiguous biphenotypic tumors, aligning predictions with underlying genetics and clinical outcomes (163).

Cross-platform and histology-based surrogates: To broaden applicability to standard clinical workflows, models that infer spatial molecular patterns from routine H&E or targeted IHC (histology-to-molecular inference) can extend Spatial AI to archival specimens and reduce assay cost and complexity (149). Cross-platform validation showing concordance between Visium, CosMx/CODEX, IMC, and histology-derived surrogates is an essential step toward regulatory acceptance.

Path2Space predicts >4,300 gene expressions directly from routine slides and identifies spatially grounded breast-cancer subgroups with distinct survival outcomes, outperforming sequencing-based biomarkers (164). PFMSP filters histopathology patches using spatial transcriptomics, improving molecular subtype accuracy in breast cancer to ~80% (158). Such histology-to-molecular inference methods enable Spatial AI integration into clinical workflows using archival tissue (**Table 4**).

Explainability, standardization, and regulatory readiness: Model interpretability is crucial for clinician trust and regulatory review. Explainable GNNs methods and module-dissection frameworks allow mechanistic tying of predictions to tissue architecture and candidate interventions (150). Clinical pipelines must also include standard operating procedures for tissue acquisition, fixation, multiplex assay QC, and harmonized computational pre-processing to ensure reproducibility across institutions (149) (**Table 4**).

#### 5.3.1 Defining surpassing conventional assays: evidence standards and pathologist collaboration

Claims that Spatial AI biomarkers surpass conventional assays require clear operational definitions specifying which endpoints are improved, in what clinical settings, under which validation standards, and where expert human interpretation remains essential.

Where pathologists excel: Anatomic pathologists possess irreplaceable expertise in tissue interpretation. Their proficiency extends to nuanced recognition of morphologic architectures, cytologic atypia, immune infiltration and exclusion patterns, stromal barriers, and TLS maturation. Beyond image evaluation, they synthesize morphologic findings with clinical history, laboratory data, and prior specimens to deliver context-specific diagnoses. Pathologists also excel at identifying artifacts, rare variants, and suboptimal tissue quality that may confound algorithmic pipelines. Through biological plausibility filtering, they discern and dismiss computational outputs inconsistent with established pathobiology or technical soundness (165).

Where Spatial AI adds measurable value: Spatial AI offers quantification capabilities that complement and extend human analysis, particularly for features that are subjective or unfeasible to assess manually. These include precision measurement of cell–cell distances, spatial neighborhood composition, graph-based connectivity, and TLS structural maturity. AI enables reproducible quantification of immune–tumor interactions, density ratios, suppressive microenvironmental clusters, and vascular–lymphoid organization. Furthermore, it facilitates high-dimensional multimodal integration across spatial transcriptomics, multiplexed proteomics, and histomorphology domains that exceed human cognitive capacity for simultaneous interpretation. Following rigorous validation, AI allows fully standardized and reproducible scoring across institutions, mitigating inter-observer variability in assays such as PD-L1 IHC or immune scoring. Transfer learning extends these benefits to rare malignancies. Importantly, AI can detect subtle and biologically relevant spatial motifs such as complex stromal–immune geometries or metabolic gradients that typically escape human observation.

Quantifying the evidence: To substantiate claims of superiority, studies must clearly define evaluation endpoints (predictive AUC, survival stratification, concordance with orthogonal biomarkers), specify cohort characteristics, and apply transparent validation strategies. To date, most reports demonstrate retrospective gains over single-marker IHC or visually derived scores; however, prospective, multi-institutional validations with predefined endpoints remain scarce. Rigorous benchmarking against clinically relevant comparators, including pathologist scoring, single-marker IHC, bulk genomic readouts, and multiplex biomarker panels, is essential. Statistical improvements should be differentiated from clinically actionable enhancements that meaningfully influence therapeutic decision-making.

Current state of evidence: As of 2025, the evidence base remains primarily composed of retrospective proof-of-concept studies, with minimal prospective validation and no Spatial AI biomarker yet cleared by major regulatory authorities for immunotherapy guidance.

#### 5.3.2 Clinical implementation roadmap

Translating Spatial AI into routine pathology requires standardized workflows that integrate within existing clinical infrastructure while addressing the practical barriers of cost, reproducibility, regulatory compliance, and comparison with established biomarkers. To support stepwise adoption, we propose a three-tier implementation model aligned with current technological maturity and institutional capabilities.

Tier 1-Research centers: At present, research centers can fully implement spatial transcriptomic and proteomic profiling integrated with GPU-accelerated GNNs analysis. This tier represents the discovery stage, enabling biomarker identification, mechanism elucidation, and stratification in clinical trials. Turnaround time generally spans 7–10 days, reflecting the complexity and scale of current platforms. A critical delivery at this tier is a head-to-head comparison of Spatial AI signatures against established clinical biomarkers, PD-L1 immunohistochemistry, tumor mutational burden (TMB), and microsatellite instability (MSI), using pre-specified endpoints in annotated trial cohorts. Early data suggest that spatial topological features predict immunotherapy response independently of PD-L1 IHC in NSCLC retrospective cohorts, though prospective validation against these standards remains the essential next step before clinical adoption can be justified (166). Tier 1 serves as the core engine for spatial biomarker development and methodological validation (167).

Tier 2-Academic medical centers: Within the next few years, targeted multiplex imaging covering approximately 30–50 markers, combined with cloud-based AI inference, is projected to become feasible in academic hospitals. These assays will focus on validated spatial biomarkers such as CAF barrier density, TLS maturity, and immune exclusion indices (53). For realistic clinical workflow integration, tissue must be processable within standard pathology turnaround times of 3–5 days, results must be deliverable in structured reports interpretable by oncologists without specialist computational training, and quality control metrics must be embedded within the assay pipeline to flag suboptimal specimens before analysis. Reproducibility barriers at this tier are substantial: inter-site variability in antibody titration, imaging acquisition, and computational preprocessing must be controlled through standardized SOPs, and cross-site concordance should be formally demonstrated before deployment in treatment-guiding roles. Training programs for pathologists in spatial report interpretation and standardized quality-control measures are essential prerequisites. Several academic centers are already piloting such workflows within ongoing immunotherapy trials, demonstrating growing readiness for translation.

Tier 3-Community hospitals: In the longer term, community hospitals are expected to adopt H&E-based surrogate models such as Path2Space or histology-to-transcriptome inference capable of inferring spatial molecular features directly from routine histopathology slides using large foundation models. These models can generate spatially resolved molecular insights with rapid turnaround of under 24 hours, offering a scalable and accessible path toward Spatial AI–enabled diagnostics. At this tier, the key practical question is whether spatial AI adds clinically actionable information beyond what PD-L1 IHC and TMB already provide in routine settings. For spatial biomarkers to justify adoption at the community level, they must demonstrate incremental predictive value over current standard assays, be deliverable at comparable or justifiable cost, and produce outputs that directly inform treatment selection rather than merely refine risk stratification. Rigorous validation against direct spatial profiling in large, paired cohorts will be essential before clinical deployment to ensure reproducibility and generalizability (168).

Regulatory pathway: Spatial AI biomarkers are regulated as software as a medical device (SaMD) within both the FDA and EMA frameworks. Under the FDA’s predetermined change control plan, developers must prospectively define key elements, including training data curation protocols, model retraining triggers and validation strategies, real-time performance monitoring approaches, and demographic and genomic bias evaluation (169). A critical and currently unresolved regulatory barrier is the absence of agreed reference standards for spatial biomarker performance, unlike PD-L1 IHC, which has defined scoring systems and companion diagnostic approvals; Spatial AI models lack standardized cutoffs, locked algorithm versions approved for specific indications, and post-market surveillance frameworks. Transparency in algorithm versioning and interpretability of decision logic are fundamental prerequisites for regulatory approval and clinical acceptance.

Benchmark comparison with established biomarkers: For clinical integration, Spatial AI biomarkers must demonstrate advantages over current molecular and immunohistochemical standards in practical settings, not only in retrospective discovery cohorts. In NSCLC treated with anti-PD-1 therapy, PD-L1 IHC achieves modest predictive performance with well-documented inter-observer variability, and TMB provides complementary but imperfect stratification. Early Spatial AI models have achieved substantially higher predictive performance in retrospective cohorts, though these gains have not yet been demonstrated prospectively or in community settings where tissue quality, assay standardization, and computational infrastructure are more variable (170). For robust clinical translation, Spatial AI systems must achieve meaningful and reproducible improvements in prediction accuracy across institutions, demonstrate clinical utility in guiding therapeutic decisions in prospective trials, and cost structures that are justifiable relative to existing biomarker panels.

Cost-effectiveness and scalability considerations: The cost of spatial omics assays currently represents a significant barrier to routine deployment. Sequencing-based spatial transcriptomics platforms cost substantially more per sample than standard IHC, and multiplexed imaging adds further reagent, instrument, and computational costs. Long-term integration of Spatial AI is expected to enhance healthcare efficiency by minimizing ineffective therapies and enabling precise treatment selection, but this value proposition requires formal health economic modelling in specific clinical contexts, for example, first-line immunotherapy selection in NSCLC or neoadjuvant therapy planning in triple-negative breast cancer, before payers and health systems will consider reimbursement. Tier 2 and Tier 3 implementations offer the highest scalability potential once validation and automation milestones are met. Cloud-based inference, containerized workflows, and foundation model fine-tuning reduce the per-sample computational cost substantially, but reagent and imaging costs remain the dominant barrier at scale. A realistic near-term path is the development of simplified targeted panels of 10–20 spatially validated markers that capture the most predictive topological features at a cost comparable to existing multiplex IHC panels, rather than full spatial transcriptomic profiling for every patient.

### 5.4 Future directions

Longitudinal spatial profiling: Time-series spatial data will enable dynamic models of TME evolution, revealing the emergence of adaptive resistance niches and informing timing and combination strategies.

Patient-specific topological interventions: Precision therapy guided by a patient’s unique spatial blueprint, such as localized ECM modulation, intratumoral delivery to immune-cold cores, or TLS-induction, represents a practical road to personalized spatial immunotherapy.

Regulatory and operational frameworks: The field must converge on validated spatial-omics assays, benchmark datasets, and regulatory endpoints that connect spatial topologies to clinically meaningful outcomes.

## 6 Challenges, limitations, and future directions

Clinical adoption of Spatial AI in oncology faces major limitations in data standardization, computational modeling, interpretability, and regulatory validation. It is important to note that the majority of Spatial AI findings presented in this review represent computational associations and retrospective correlations; prospective experimental validation and clinical trials are required before causal claims can be made or therapeutic decisions guided by these models. Addressing these interdependent barriers will be essential to unlock its full potential for precision immunotherapy.

### 6.1 Data standardization, batch effects, and reproducibility

Batch effects and technical variability often dominate true biological signals. Differences in tissue fixation, sequencing depth, imaging exposure, and antibody composition can bias downstream analyses (171). Conventional normalization techniques, such as mutual nearest-neighbor (MNN) correction and Harmony, offer only partial solutions (172). In contrast, newer adversarial domain-adaptation and graph-based normalization approaches better preserve spatial topology and mitigate platform-specific artifacts (173). Establishing uniform benchmarking datasets and quantitative metrics remains essential for cross-laboratory evaluation.

Computational scalability is an emerging challenge as high-resolution spatial datasets reach terabyte scale, demanding distributed computing, GPU acceleration, and optimized data tiling. Frameworks such as scvi-tools, MONAI, and GraphOmics enable scalable multimodal analysis (16), yet model training, interpretability, and data visualization remain computationally intensive. Recent advances in graph representations and sparse attention mechanisms improve scalability without sacrificing spatial fidelity or biological resolution.

Interpretability is critical for clinical translation. Deep-learning models, especially graph neural networks (GNNs), often function as opaque “black boxes,” limiting clinician trust and regulatory approval. Methods such as GNNExplainer and GraphSHAP enhance transparency by identifying influential subgraphs and molecular drivers of model predictions. Incorporating biologically constrained priors such as ligand–receptor interaction networks and curated signaling pathways further increases mechanistic interpretability and enables hypothesis-driven biomarker discovery.

Finally, regulatory validation represents the last frontier. Most Spatial AI biomarkers remain at the retrospective validation stage. Prospective, multi-institutional studies integrating spatial signatures into diagnostic and therapeutic workflows are crucial to demonstrate reproducibility and clinical utility. Regulatory bodies increasingly emphasize transparency, data provenance, and standardized reporting frameworks. Initiatives such as the Spatial Biomarker Consortium and the FDA Digital Health Center of Excellence are now establishing guidelines for spatial biomarker evaluation and clinical deployment (174).

Addressing data heterogeneity in spatial omics requires coordinated standardization across technical, computational, and consortium levels. We propose a multilevel harmonization strategy encompassing experimental protocols, computational normalization, benchmarking, and metadata governance.

Technical standardization: Standardizing tissue processing protocols across institutions is essential for reproducibility and cross-cohort integration. Uniform fixation conditions (24–48 h in 10% neutral-buffered formalin for FFPE tissues), consistent section thickness (4–5 μm for spatial transcriptomics, 5–10 μm for multiplexed imaging), and controlled environmental parameters (temperature and humidity) during sectioning are critical for data comparability. The Standard Operating Procedures (SOPs) established by the Human Tumor Atlas Network (HTAN) provide a foundational reference that should be broadly adopted. Pre-analytical quality metrics, including the RNA Integrity Number (RIN) for fresh-frozen samples and DV200 for FFPE tissues, should be routinely reported to ensure transparency in sample quality across spatial transcriptomics datasets (175).

Computational normalization pipelines: We recommend a three-tier normalization framework to mitigate technical variability while preserving spatial context. The first stage involves within-sample normalization through sequencing depth adjustment (library size scaling, quantile normalization, or SCTransform for spatial data). The second stage applies batch correction methods that retain spatial topology, such as Harmony on spatially aware principal components or mutual nearest neighbor (MNN) alignment in a latent spatial space instead of the raw gene space. The third stage incorporates domain-adversarial training within deep learning frameworks, in which an auxiliary classifier predicts batch identity from learned embeddings and the primary network is trained to minimize this predictability, thereby producing batch-invariant yet biologically meaningful representations (21).

Benchmark datasets and evaluation metrics: Field-wide progress depends on the establishment of standardized benchmark datasets with well-annotated ground truth. These resources should include matched serial tissue sections analyzed across multiple spatial platforms from identical tumor specimens to enable direct cross-platform comparisons. Benchmark evaluation should employ unified metrics: spatial fidelity (Moran’s I, Geary’s C), biological signal retention (correlation between canonical marker expression and annotated cell types), and cross-platform concordance (Spearman correlation of gene signatures, spatial domain overlap). We advocate for an annual community benchmarking initiative for spatial omics, analogous to CASP in structural biology or ImageNet challenges in computer vision, to drive reproducibility and methodological advancement (176).

Metadata standards and data sharing infrastructure: Robust Metadata and open data sharing are vital for reproducibility and secondary analysis. All spatial omics datasets should be deposited in public repositories (GEO, SRA, Zenodo), accompanied by standardized metadata following spatial extensions of MIAME principles. Required metadata should capture sample provenance, technical parameters, and quality control metrics (spot or cell counts, transcript detection rate, signal-to-noise ratio). Adoption of the SpatialData framework will ensure interoperable, machine-readable data structures across analysis platforms and facilitate seamless integration in downstream computational pipelines (171).

### 6.2 The imperative of explainable AI in clinical decision-making

Clinical adoption of Spatial AI requires transparency and interpretability to build trust and meet regulatory standards. Clinicians must understand why an algorithm predicts response or resistance to immunotherapy. Explainable AI (XAI) techniques for graph neural networks (GNNs), such as Graph Attention Networks (GATs) and subgraph masking, can highlight key spatial interactions and topological features that drive predictions (124, 141).

Bias remains a critical concern. Models trained on limited or homogeneous cohorts risk encoding racial or genomic biases. Continuous auditing with XAI tools enables fairness assessment and ensures consistent performance across diverse patient populations (177).

Regulatory agencies, including the FDA and EMA, emphasize interpretability as essential for analytical validation and approval of AI-based diagnostics (178). Transparent models with traceable decision pathways will be central to establishing clinical credibility and regulatory compliance. Thus, XAI is not optional but fundamental, linking biological insight, clinical trust, and ethical deployment of Spatial AI in oncology.

### 6.3 Computational bottlenecks and scalability

Spatial omics technologies generate massive, high-dimensional datasets, spanning single-cell transcriptomes to gigapixel tissue images, far exceeding the capacity of conventional pipelines. Graph construction and model training remain major bottlenecks. Whole-slide images at single-cell resolution can yield millions of nodes, making GNNs training computationally intensive. Advances in graph pooling, sparse representations, and distributed GPU/TPU pipelines are crucial for scaling Spatial AI to clinically relevant cohort sizes (179).

Multi-scale integration is another challenge. The tumor microenvironment (TME) operates across subcellular, cellular, and tissue-level hierarchies. Multi-scale architectures that fuse molecular features with histomorphological context are needed to preserve both local and global topology, enabling mechanistic modeling of immune tumor interactions (180, 181).

Current assays largely capture 2D tissue sections, limiting spatial fidelity. 3D reconstruction of the TME using serial imaging, light-sheet microscopy, or volumetric modeling combined with AI-based spatial registration offers a path to resolving complex immune niches such as tertiary lymphoid structures (182).

Achieving clinical-grade scalability in Spatial AI for gigapixel whole-slide images containing millions of cells demands coordinated advances in algorithm design, distributed computing, and deployment infrastructure.

Graph coarsening and hierarchical pooling: To handle ultra-large spatial graphs, hierarchical representations should be constructed where fine-grained nodes represent individual cells and successive levels aggregate spatially proximal groups into super-nodes (183). This multi-resolution design enables GNNs to learn cellular-level patterns at lower tiers and capture tissue-scale organization at higher tiers.

Sparse attention and efficient transformers: The quadratic complexity of standard transformer attention is infeasible for whole-slide contexts. Sparse attention mechanisms that restrict patch interactions to spatially neighboring regions reduce complexity to linear scaling, while approximations like Linformer and Performer further accelerate global attention computations. For spatial omics, a hybrid attention strategy local attention within tissue subregions combined with global attention across representative patches balances biological fidelity with computational efficiency.

Distributed and federated learning: Large models require parallelized training across multi-GPU or multi-node systems. Data parallelism distributes distinct image tiles across GPUs with synchronized gradient updates, whereas gradient checkpointing conserves memory by recomputing activations during backpropagation. For multi-institutional studies under data privacy constraints, federated learning allows decentralized model training where only parameters, not raw data, are shared. This enables collaborative model development while maintaining patient confidentiality (184).

Cloud infrastructure and containerization: Cloud-based implementations on platforms such as AWS, Google Cloud, or Azure facilitate elastic scaling of GPU clusters for Spatial AI workloads. Workflow containerization via Docker and orchestration through Kubernetes ensures reproducibility and portability across institutions. Hosting pre-trained foundation models on public model repositories (Hugging Face, TorchHub) with version control and standardized documentation further democratizes access to state-of-the-art architectures, reducing dependence on local high-performance computing resources (185).

Approximation methods for real-time inference: Clinical deployment requires near real-time inference, ideally under 10 minutes per slide. Model compression techniques such as quantization (reducing precision from 32-bit to 8-bit), pruning (removal of low-saliency parameters), and knowledge distillation (training lightweight student models to emulate larger teachers) can reduce model size by 4–10× and accelerate inference by 3–5× with minimal performance loss. Together, these approximations make real-time, clinically deployable Spatial AI feasible without sacrificing interpretability or diagnostic accuracy (186).

### 6.4 Translational and regulatory challenges

For Spatial AI to influence clinical care, predictive spatial signatures must undergo rigorous validation and meet regulatory standards.

Validation pipelines should follow a stepwise framework including discovery in high-plex spatial datasets, independent validation across multi-institutional cohorts, and translation into clinically actionable assays, such as simplified mIF or targeted imaging panels (4).

Prospective clinical trials are essential to establish the predictive utility of Spatial AI biomarkers relative to current standards like PD-L1 immunohistochemistry or tumor mutational burden. Incorporating spatial topology into trial design may enable adaptive therapy strategies and rational selection of combinatorial immunotherapies (187).

Regulatory approval and quality control require demonstration of both analytical and clinical validity, alongside robust maintenance of model performance over time despite software updates. Transparent algorithm-change protocols, adherence to FDA/EMA guidelines, and reproducible reporting frameworks are critical for clinical adoption (188).

Regulatory approval of Spatial AI biomarkers requires structured evidence spanning analytical validation, clinical validation, prospective evaluation, and post-market monitoring (189, 190).

Analytical validation: Analytical validation confirms that the AI model produces accurate, reproducible results. Reference materials such as tissue microarrays representing known immune phenotypes (inflamed, excluded, desert) should be scored by expert pathologists to establish ground truth. Key metrics include inter-run precision (coefficient of variation < 15%), inter-site reproducibility (concordance correlation coefficient > 0.90), and detection limits defined by minimum quantifiable cell densities or transcript counts. These studies define the assay’s technical reliability and performance range.

Clinical validation: Clinical validation assesses whether the biomarker provides actionable clinical value. Retrospective–prospective studies using archived trial specimens with outcome data should follow pre-specified analysis plans, including endpoint definitions (two-year progression-free survival), performance benchmarks (C-index > 0.70), and comparison to standard biomarkers such as PD-L1 IHC. Essential elements include blind evaluation, independent validation cohorts, and transparent reporting of all pre-planned and exploratory results to confirm generalizability.

Prospective trial integration: Integration into prospective randomized trials provides Level 1 evidence for clinical benefit. Ideal designs stratify patients by AI biomarker score and randomize to standard versus biomarker-guided therapy. Alternatively, biomarker-stratified analyses can be embedded within ongoing trials in collaboration with sponsors.

Regulatory submission Strategy: For FDA 510(k) or PMA approval, required documentation includes the locked algorithm version, full validation data, failure mode analysis, and post-market surveillance plan. Companion diagnostics must align biomarker cutoffs with therapeutic benefit. Under EU IVDR, CE-IVD marking similarly requires analytical and clinical evidence plus conformity assessment by a notified body.

Standardized reporting framework: Publications describing Spatial AI biomarkers should follow TRIPOD guidelines adapted for spatial omics. Reports must specify intended use population, model architecture, training workflow, data and code availability, subgroup-level metrics, and potential limitations or biases.

Ongoing model monitoring: Post-approval, models require continuous performance audits comparing predictions with real-world outcomes. Retraining should be triggered by > 10% performance decline in six-month rolling analyses. Version control, complete audit trails, and regulatory notification for substantive model updates are essential for compliance and clinical trust.

### 6.5 Future directions

Spatial AI offers transformative opportunities for immuno-oncology despite current limitations. Longitudinal monitoring of the tumor microenvironment during therapy can uncover mechanisms of acquired resistance and help guide adaptive treatment strategies. Integrating multi-omics data, including spatial profiles, genomics, epigenomics, metabolomics, and circulating biomarkers from liquid biopsies, may boost predictive power and clinical utility. Automated clinical workflows encompassing tissue preparation, image acquisition, AI-driven analysis, and standardized reporting could make spatial diagnostics routine in pathology laboratories. Additionally, generative AI methods enable synthetic TME modeling to simulate therapy responses, which could accelerate the development of optimized combination strategies before clinical trials or patient intervention. Together, these innovations forecast a future where Spatial AI drives personalized and actionable immunotherapy by decoding immune evasion mechanisms (191–193).

## 7 Future priorities

Spatial AI has established three foundational capabilities in cancer immunology, including quantitative mapping of immune evasion architectures, predictive biomarkers exceeding single-marker assays in retrospective cohorts, and mechanistic hypotheses linking tissue topology to immunotherapy resistance. However, the path from spatial discovery to actionable therapy remains obstructed by interconnected bottlenecks in biology, technology, standardization, and clinical validation that the field must now collectively address.

### 7.1 Critical bottlenecks blocking clinical translation

The most immediate barrier is not technological but evidential. As of 2025, no Spatial AI biomarker has achieved prospective validation in a randomized immunotherapy trial or regulatory clearance for clinical use. The evidence base remains dominated by retrospective proof-of-concept studies in which spatial signatures are associated with outcome but not yet proven to guide therapeutic decisions. Closing this gap requires embedding spatial biomarker analysis within prospective randomized trials with pre-specified analysis plans, defined clinical endpoints such as two-year progression-free survival, and direct comparison against established standards including PD-L1 IHC, TMB, and MSI. Without this Level 1 evidence, Spatial AI will remain a research tool regardless of its biological sophistication.

Reproducibility across institutions represents the second critical barrier. Spatial omics platforms, such as Visium HD, Xenium, CosMx, IMC, and CODEX, produce heterogeneous coordinate systems, spatial resolutions, and feature annotations that impede cross-site model transfer (170, 171). Batch effects from differences in tissue fixation, sequencing depth, imaging exposure, and antibody composition can dominate true biological signals (171). Adversarial domain-adaptation and graph-based normalization approaches partially mitigate these effects (173), but universal standards for spatial reference models, file formats, and pre-analytical quality metrics remain lacking. An international Spatial Omics Consortium analogous to TCGA, with open-access repositories containing 1,000 or more annotated specimens profiled across Visium, Xenium, and IMC, would provide the benchmark infrastructure needed for rigorous cross-platform validation (171). Uniform fixation conditions, section thickness standards, and mandatory reporting of RNA Integrity Number and DV200 metrics should be adopted as field-wide minimum requirements (175).

Computational accessibility remains a third practical barrier. Current GNNs pipelines require specialized expertise and substantial GPU resources that most clinical laboratories do not possess. Foundation models pre-trained on large histopathology atlases, such as the PathOmics-1M initiative and the general-purpose computational pathology model, enable fine-tuning with modest resources through transfer learning. Cloud-based inference, containerized workflows via Docker and Kubernetes, and model repositories on platforms such as Hugging Face will further democratize access, reducing dependence on local high-performance computing.

### 7.2 Most critical open biological questions

Despite substantial progress in mapping immune evasion topology, three biological questions remain unanswered and are directly limiting therapeutic translation. First, the causal relationships between spatial architecture and immune resistance are largely inferred from correlative spatial data. It remains unclear whether CAF-dense exclusion zones actively suppress T-cell infiltration or whether they reflect a pre-existing resistant state selected by tumor evolution. Spatially resolved CRISPR screening platforms now offer a path to resolve this by disrupting candidate resistance circuits *in situ* and measuring downstream changes in immune connectivity. These tools can distinguish causal drivers from correlated bystanders (194). Prioritizing these experiments for the highest-confidence Spatial AI predictions, such as FAP^+^ CAF barriers in NSCLC and TAM–Treg co-localization in HGSCC, would convert descriptive topology into validated mechanistic targets.

Second, the dynamics of spatial resistance remain poorly understood. Current spatial omics datasets are almost entirely cross-sectional, capturing a single timepoint in tumor evolution. Longitudinal spatial profiling through serial biopsies during therapy is feasible in metastatic melanoma and renal cell carcinoma and has demonstrated that adaptive resistance niches emerge on treatment in patterns invisible to bulk molecular profiling (195). Standardized protocols for serial spatial profiling in larger immunotherapy trial cohorts would reveal whether resistance architectures are pre-formed or dynamically assembled, directly informing the timing and sequencing of combination strategies.

Third, three-dimensional tissue organization is currently inaccessible to standard spatial omics workflows that capture two-dimensional sections. Complex immune structures such as tertiary lymphoid structures and vascular-immune interfaces extend across multiple tissue planes, and their functional competence may depend on three-dimensional organization invisible in single sections. Volumetric imaging approaches combined with AI-based spatial registration, exemplified by the VORTEX framework for three-dimensional spatial transcriptomics (181), offer a scalable path toward resolving these structures and understanding their full functional architecture (196).

### 7.3 The path from spatial discovery to actionable therapy

Realizing the clinical potential of Spatial AI requires a defined translational pipeline connecting discovery, validation, and deployment. At the discovery stage, sequencing-based spatial transcriptomics provides hypothesis-generating breadth across the transcriptome, identifying candidate spatial resistance signatures in well-annotated trial cohorts. Candidate signatures, effector-to-tumor path lengths, suppressive niche clustering coefficients, TLS connectivity indices, and CAF barrier density are then taken forward to targeted imaging-based validation using Xenium, CosMx, or IMC panels focused on the most predictive markers. This validation stage must include cross-platform concordance testing, inter-site reproducibility assessment, and direct comparison against PD-L1 IHC and TMB in the same cohorts.

Validated spatial biomarkers then enter prospective trial integration, where patients are stratified by Spatial AI score and randomized to standard versus biomarker-guided therapy, generating the Level 1 evidence required for regulatory submission. For FDA 510(k) or PMA approval, documentation must include the locked algorithm version, full validation data, failure mode analysis, and a post-market surveillance plan. Companion diagnostics must align biomarker cutoffs with demonstrated therapeutic benefit, and models must undergo continuous performance auditing with retraining triggered by performance decline exceeding ten percent in six-month rolling analyses.

The near-term realistic path for community deployment lies not in full spatial transcriptomics but in simplified targeted panels of ten to twenty spatially validated markers that capture the most predictive topological features at a cost comparable to existing multiplex IHC, delivered through H&E-based surrogate models such as Path2Space that infer spatial molecular features directly from routine histopathology (164). Patient-specific interventions guided by individual spatial blueprints, localized ECM modulation, intratumoral delivery to immune-cold cores, TLS induction in immune-desert regions, represent the ultimate clinical goal, translating spatial maps of resistance directly into precision combination strategies (172).

Immune evasion is fundamentally a topological problem. Spatial AI provides a principled computational framework to decode tumor microenvironment architectures and transform qualitative histopathology into quantitative, actionable biomarkers. Achieving this clinical impact demands coordinated community effort in standardization, prospective validation, mechanistic confirmation, and responsible translation, to overcome not only the molecular but the geometric barriers that limit cancer immunotherapy (21).

## 8 Conclusion

The emergence of Spatial AI, which unites high-resolution multi-modal omics with advanced Deep Learning, represents a fundamental shift in cancer therapy. Immune resistance is primarily a topological challenge, not just a molecular one, driven by the physical architecture of the tumor microenvironment. Spatial AI moves beyond conventional biomarkers by quantifying specific topological phenotypes, such as CAF-driven immune exclusion and TLS maturity, to generate predictive metrics. To achieve clinical relevance, this field must address significant challenges in data standardization, computational scalability, and Explainable AI. Ultimately, by precisely mapping the architecture of resistance, Spatial AI offers a robust strategy to develop targeted combination therapies, thus overcoming the geometric barriers that limit current cancer immunotherapy.
